# Probabilistic mapping of language networks from high frequency activity induced by direct electrical stimulation

**DOI:** 10.1002/hbm.25112

**Published:** 2020-07-22

**Authors:** Marcela Perrone‐Bertolotti, Sarah Alexandre, Anne‐Sophie Jobb, Luca De Palma, Monica Baciu, Marie‐Pierre Mairesse, Dominique Hoffmann, Lorella Minotti, Philippe Kahane, Olivier David

**Affiliations:** ^1^ CNRC, Laboratoire de Psychologie et NeuroCognition University of Grenoble Alpes, University of Savoie Mont Blanc Grenoble France; ^2^ Institut Universitaire de Paris France; ^3^ CHU Grenoble Alpes, Pôle Neurologie Psychiatrie Grenoble France; ^4^ University of Grenoble Alpes, Grenoble Institut Neurosciences, GIN Grenoble France; ^5^ Inserm Grenoble France; ^6^ CHU Grenoble Alpes, Pôle Tête et Cou Grenoble France

**Keywords:** direct electrical stimulation, high frequency activity, intracranial electroencephalography, language, naming, paraphasia, speech arrest

## Abstract

Direct electrical stimulation (DES) at 50 Hz is used as a gold standard to map cognitive functions but little is known about its ability to map large‐scale networks and specific subnetwork. In the present study, we aim to propose a new methodological approach to evaluate the specific hypothesis suggesting that language errors/dysfunction induced by DES are the result of large‐scale network modification rather than of a single cortical region, which explains that similar language symptoms may be observed after stimulation of different cortical regions belonging to this network. We retrospectively examined 29 patients suffering from focal drug‐resistant epilepsy who benefitted from stereo‐electroencephalographic (SEEG) exploration and exhibited language symptoms during a naming task following 50 Hz DES. We assessed the large‐scale language network correlated with behavioral DES‐induced responses (naming errors) by quantifying DES‐induced changes in high frequency activity (HFA, 70–150 Hz) outside the stimulated cortical region. We developed a probabilistic approach to report the spatial pattern of HFA modulations during DES‐induced language errors. Similarly, we mapped the pattern of after‐discharges (3–35 Hz) occurring after DES. HFA modulations concurrent to language symptoms revealed a brain network similar to our current knowledge of language gathered from standard brain mapping. In addition, specific subnetworks could be identified within the global language network, related to different language processes, generally described in relation to the classical language regions. Spatial patterns of after‐discharges were similar to HFA induced during DES. Our results suggest that this new methodological DES‐HFA mapping is a relevant approach to map functional networks during SEEG explorations, which would allow to shift from “local” to “network” perspectives.

## Graphical abstract

We adopted a new probabilistic brain mapping approach based on direct cortical stimulation and high frequency activity measures during naming task. Our results suggest that this new methodological direct electrical stimulation‐high frequency activity mapping is a relevant approach to map functional networks during stereo‐electroencephalographic explorations, which would allow to shift from “local” to “network” perspectives.

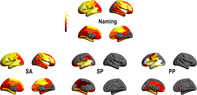

## 1 INTRODUCTION

Direct electrical stimulation (DES) has been for a long time a routine clinical practice during intraoperative and extraoperative neurological evaluation (Penfield & Jasper, 1954). Extraoperative evaluation is specifically performed during presurgical stereo‐electroencephalography (SEEG) evaluation in patients with severe drug‐resistant epilepsy, candidates to curative resective surgery. DES is performed in these patients for two main objectives (Kahane et al., 1993; Kahane & Dubeau, 2014; Penfield & Jasper, 1954): (a) spatial definition of the “epileptogenic zone” (Rosenow & Lüders, 2001) and surgical boundaries by attempting to reproduce the patient's usual type of seizure, and (b) spatial identification of “functional zones” (i.e., essential cortex) to be spared during surgery in relation to cognitive functions such as language. DES is currently considered as a “gold‐standard” method for individual functional mapping for both intraoperative and extraoperative evaluations (Desmurget, Song, Mottolese, & Sirigu, 2013; Mandonnet, Winkler, & Duffau, 2010). In this context, when a small electrical current is delivered in one cortical site and a transient functional disruption is observed (e.g., speech arrest [SA]), this region is considered to be an eloquent area. Thus, given the causality between electrical stimulation and behavior deficit or dysfunction, DES is used to predict potential deficits during cortical resection and the decision to perform the resective surgery is based on its results (for recent reviews of DES, see Borchers, Himmelbach, Logothetis, & Karnath, 2011; Desmurget et al., 2013; Selimbeyoglu & Parvizi, 2010).

It has been shown that a cortical site stimulation could induce different behavioral responses and a similar behavioral response (e.g., SA) can result from the stimulation of various sites (Borchers et al., 2011; Desmurget et al., 2013). This is in line with the current view considering that cognitive functions brain representation depends on large brain networks (Power et al., 2011; Yeo et al., 2011), that is, distributed groups of interconnected and synchronized neurons, rather than isolated functional cortical areas (Duffau, Gatignol, Mandonnet, Capelle, & Taillandier, 2008; Duffau, Moritz‐Gasser, & Mandonnet, 2014). Thus, during DES, an specific language dysfunction could be observed by the stimulation of different cortical site of a specific subnetwork (e.g., phonological or semantic paraphasia [SP]) (Mandonnet et al., 2010; Mandonnet et al., 2016). This is also in agreement with results indicating that over 50% of patients show postoperative language deficits (naming decline) despite DES for language mapping prior to surgery (Davies, Risse, & Gates, 2005). Therefore, functional deficit induced by 50 Hz DES of a cortical region may reflect dysfunction of a large‐scale network (Mandonnet et al., 2010; Mandonnet et al., 2016). For instance, in a case study, DES on basal temporal cortex (BTC) produced aphasic symptoms without language deficits after BTC resection (Ishitobi et al., 2000). This was explained by the association during the DES on BTC with intrastimulus remote discharges in posterior superior temporal cortex and thus suggested that distant effect outside the stimulating current field is exerted by DES (Ishitobi et al., 2000). Thus, intrastimulus remote discharges as well as after‐discharges can result in false localization of functional cortex as they may occur remotely from the stimulate site and consequently induce behavioral manifestation unrepresentative of the stimulate cortical site (Blume, Jones, & Pathak, 2004; Karakis et al., 2015). In the present study, we hypothesize that DES‐induced language interference (naming errors) could be considered as input gates into a larger language network (Mandonnet et al., 2010).

For the present study, we developed a new approach to map the networks showing high frequency activities (HFAs) induced by 50 Hz DES during language disturbances. The method is largely inspired by the one already developed by our group to map ictal HFAs (David et al., 2011). Specifically, this methodology aims to identify brain areas whose HFA is significantly greater than baseline HFA using standard neuroimaging time series analysis. This involves a simple categorical comparison (using *t* tests) between mean activity at baseline and mean activity over short windows (e.g., 3 s) at various times (e.g., 0–20 s) after DES. The significance of these differences was evaluated in relation to the variability of fluctuations within each time segment. The data features we compared were the fluctuations in HFA (70–150 Hz), shown to be a potential specific biological support of cognitive function (Lachaux, Axmacher, Mormann, Halgren, & Crone, 2012; Muller et al., 2018; Perrone‐Bertolotti et al., 2020). We applied this methodology to assess large‐scale language networks by evaluated DES‐induced language errors and measured HFA modification during these errors in nonstimulated brain regions. According to our working hypothesis, each cortical site in which DES induces language errors may be part of a larger language network, in which the activity is also modulated by DES (Mandonnet et al., 2010). Therefore, language errors induced by the DES may reflect activity perturbation of a large‐scale language network instead of only the stimulated site and would explain why similar language symptoms are associated with electrical disturbances not only of local but also of remote cerebral regions (Mulak, Kahane, Hoffmann, Minotti, & Bonaz, 2008). Consequently, this methodological approach can be able to identify language network and subnetworks reflected by the HFAs cooccurrence, in either adjacent or distant regions of the stimulation site, and in particular in perisylvian and extrasylvian language areas.

To address our objective, we retrospectively analyzed data from extraoperative DES performed in 29 patients with drug‐resistant epilepsy. For each patient, we identified the language errors induced by the DES during a picture‐naming task and reported the corresponding stimulation cortical sites. This task, classically used during clinical DES language mapping, involves a large fronto‐temporo‐parietal network (Corina et al., 2010; Haglund, Berger, Shamseldin, Lettich, & Ojemann, 1994; Indefrey & Levelt, 2004; Ojemann, 1983; Rofes et al., 2018). First, we mapped the HFA elicited in nonstimulated recorded sites during each observed type of language error. Second, we put in relation the HFA modifications with five classical core language regions in order to identify language subnetworks related to linguistic processes underlying language errors induced by the DES during naming.

## 2 MATERIALS AND METHODS

### 2.1 Patients

From the archives of Grenoble University SEEG laboratory from 2009 to 2013, we identified 29 patients (13 females) who carried out standard presurgical evaluations and experienced language symptoms elicited by DES (Table 1). Patients were fully informed and gave their consent to undergo SEEG recordings as part of the presurgical evaluation of their drug‐resistant epilepsy, in addition to noninvasive exams (high‐resolution structural magnetic resonance imaging [MRI], video‐EEG monitoring, neuropsychological evaluation). DES and SEEG recordings were performed according to the routine procedure used at Grenoble University Hospital (Kahane et al., 1993; Kahane & Dubeau, 2014).

**TABLE 1:** Demographic and clinical data of Patients P1–P29

P	S	A	H	SO	LL	IL	MRI	SL	Other	Engel scale	VCI/POI
P1	M	20	R	6	LH	LH	L lenticulo‐insular scar	L insulo‐temporal (T1)	NS	IV	NA
P2	M	12	R	11	LH	RH	N	R frontal premotor (F1l)	FCD II	IA	109/106
P3	M	24	R	9	LH	RH	N	R prefrontal (anterior)	FCD I	IA	NA/97
P4	M	20	R	NA	LH	LH	N	No surgery*	NA	NA	NA
P5	M	33	R	12	LH	LH	N	L temporal (antero‐mesial)	NS	IIA	NA
P6	M	22	R	12	LH	B	L mesial occipital tumor	L occipital (mesial)	FCD I	IIA	127/117
P7	F	35	L	10	LH	RH	R parietal postoperative scar	R frontal premotor (F2)	NS	IV	92/83
P8	F	50	R	11	LH	LH	L motor opercular tumor	L motor opercular	FCD II	III	89/80
P9	F	41	R	8	LH	LH	Left fronto‐opercular FCD	Left fronto‐opercular	FCD II	IA	99/119
P10	F	16	R	5	LH	LH	F frontal premotor FCD	L frontal premotor (F1)	FCD II	ID	NA
P11	M	42	R	17	LH	LH	N	L temporal (antero‐mesial)	NS	IVA	131/112
P12	F	36	R	5	LH	LH	L occipito‐temporal postoperative scar	L occipito‐temporal (basal)	FCD I	IV	NA
P13	F	26	R	14	LH	LH	N	L premotor (SMA)	FCD II	IA	83/81
P14	M	32	R	NA	LH	B	N	No surgery**	NA	NA	90/100
P15	M	26	R	17	LH	LH	N	L temporal (antero‐mesial)	NS	IA	86/106
P16	F	30	R	20	LH	LH	N	L temporal (antero‐mesial)	NS	IA	120/110
P17	M	30	R	13	RH	LH	L perisylvain polimicrogyria	Left temporo‐parieto‐occipital disconnection	NA	IA	NA
P18	F	13	R	0.25	LH	LH	Corpus callosum agenesis	L premotor (F1‐F2)	EI	IVA	79/54
P19	M	21	R	NA	LH	B	N	No surgery**	NA	NA	NA
P20	M	43	R	13	LH	LH	L temporal arachnoid cyst	L antero‐mesial	FCD I	IIA	NA
P21	F	29	R	14	LH	LH	N	No surgery***	NA	NA	94/74
P22	M	24	R	NA	LH	LH	L perisylvian atrophy	L parieto‐opercular RFTC	NA	NA	92/79
P23	M	24	R	9	LH	LH	N	L prefrontal (anterior)	NS	IIB	NA
P24	M	55	R	22	LH	RH	R temporal scar	R insular RFTC	NA	IA	129/99
P25	F	39	R	30	LH	LH	N	L temporal (basal)	FCD I	IIIA	NA
P26	F	35	R	19	LH	LH	R temporal cyst	L temporal (antero‐mesial)	FCD I	IIA	NA
P27	F	27	L	14	LH	LH	Left thalamic infarct	L posterior cingulate	NS	IB	99/76
P28	F	31	R	18	LH	LH	N	L temporal (antero‐mesial)	NA	IIC	109/89
P29	M	56	R	27	LH	LH	L temporo‐basal FCD	L temporal (basal)	FCD III	IID	NA/1

*Note:* Sex –S‐ (male, M; female, F); age at data acquisition –(a); handedness –H‐ (right‐handed, R; left‐handed, L); age seizure onset –SO‐; fMRI hemispheric lateralization for language –LL‐ (left hemisphere, LH; right hemisphere, RH; bilateral, (b); SEEG implantation hemisphere lateralization –IL‐; anatomical MRI lesion –MRI‐; surgery location –SL‐; other clinical relevant information –other‐; Engel scale; neuropsychological scores: VCI and perceptual organization index

Abbreviations: EI, eosinophilic inclusions; F, first frontal gyrus; F2, second frontal gyrus; FCD I/II, focal cortical dysplasia Type I/II; H, hemisphere; HS, hippocampal sclerosis; L, left; MRI, magnetic resonance imaging; N, normal; NA, not applicable; NS, nonspecific; R, right; RFTC, radio‐frequency thermo‐coagulation; SEEG, stereo‐electroencephalographic; SMA, supplementary motor area; T1, first temporal gyrus; VCI, verbal comprehension index.

*Multifocal epilepsy; **bifrontal epilepsy; ***bitemporal epilepsy.

### 2.2 Electrode implantation and positioning

A total of 11–15 semirigid, multilead electrodes were stereotactically implanted in each patient. Each electrode had a diameter of 0.8 mm and, depending on the target structure, consisted of 8–18 contact leads 2 mm wide and 1.5 mm apart (DIXI Medical, Besançon, France). Electrodes implantation was strictly related with individual clinical hypothesis. A preoperative MRI and postoperative MRI or CT scan were co‐registered to assess the locations of the electrode contacts for each patient using a coordinate system in relation to the anterior commissure/posterior commissure plane. Electrode contact positions were finally expressed in the Montreal Neurological Institute (MNI) coordinate system to allow group analyses after brain spatial normalization using Statistical Parametric Mapping 12 software (SPM12, Wellcome Department of Imaging Neuroscience, University College London, www.fil.ion.ucl.ac.uk/spm). Visual inspection of the contact locations was also used to check whether each electrode contact was located in gray or white matter. Furthermore, to be able to perform group analysis, we used regions of interest (ROIs) in the automated anatomical labeling (AAL) parcellation system (Tzourio‐Mazoyer et al., 2002), including 43 cortical regions per hemisphere being potentially implantable with SEEG electrodes.

### 2.3 SEEG recordings

SEEG recordings were performed using a video‐EEG monitoring system (Micromed, Treviso, Italy) that allowed to simultaneously record up to 256 monopolar contacts, so that a large range of mesial and cortical areas, as well as fissural cortices, was sampled for each patient. Sampling rate was 512 Hz, with an acquisition band‐pass filter between 0.1 and 200 Hz. Data were acquired using a referential montage with reference electrode chosen in the white matter. All other recording sites were chosen in the gray matter. For data analysis, we used a bipolar montage between adjacent contacts of the same electrode to improve sensitivity to local current generators. Coordinates of virtual bipolar contacts that were used to construct images (see below) were chosen to be at an equal distance of two successive contacts.

### 2.4 Direct electrical stimulation

Stimulations at 50 Hz were applied between two contiguous contacts at different levels along the axis of each electrode, chosen to be in the gray matter according to visual inspection of spontaneous SEEG signals. A bipolar montage was used to help eliminate the signal common to adjacent electrode contacts and to avoid artifacts coming from distant sources. Bipolar stimuli were delivered using a repetitive biphasic square wave electric currents of 1–3 ms pulse width designed for a safe diagnostic stimulation of the human brain (Micromed), according to parameters proved to produce no structural damage. The intensity used was usually 3 mA or less (down to 1 mA), for a maximum duration of 5 s, or less depending on the type of the induced clinical response.

### 2.5 Picture naming

Patients were required, in others, to overtly perform a picture‐naming task in black and white drawing images representing objects from several semantic categories (Snodgrass & Vanderwart, 1980). Prior to DES, patients were trained to name each picture (control trials) in order to ensure task feasibility and picture familiarity (behavioral baseline level). During DES, pictures were presented randomly by the neurologist and the stimulation was concomitantly applied. Naming errors were considered only when the behavioral performance was disrupted during DES compared to the reference condition (control trials without DES) performances. It is important to note that patients also performed other tasks (such as reading) for the clinical purpose of functional explorations, not presented here. However, the reading errors induced by stimulations were used to control for the specialization of language areas in naming (see Section 3.2).

### 2.6 Types of naming errors

Naming errors were retrospectively analyzed for each patient by a speech therapist according to other previous DES studies using picture naming (Corina et al., 2010). Three types of naming errors have been identified: (a) SA: with total or partial lack of naming, including anomia, speed reduction, and hypophonia (soft speech); (b) SP including word substitution with a related or associated word (i.e., the word “clock” when the picture presented represented a “watch” or the word “animal” for the target word “dog”); (c) phonological paraphasia (PP) including words errors related to phonological retrieval and phoneme selection (e.g., phonemic omission, substitutions, elision, transposition or inversion) in which a nonreal word with a phonological resemblance to the target word was generated (e.g., for the target word “pencil” the word “bencil” is generated).

### 2.7 Data processing and statistical analysis

#### 2.7.1 Review of SEEG/video events

**FIGURE 1:**
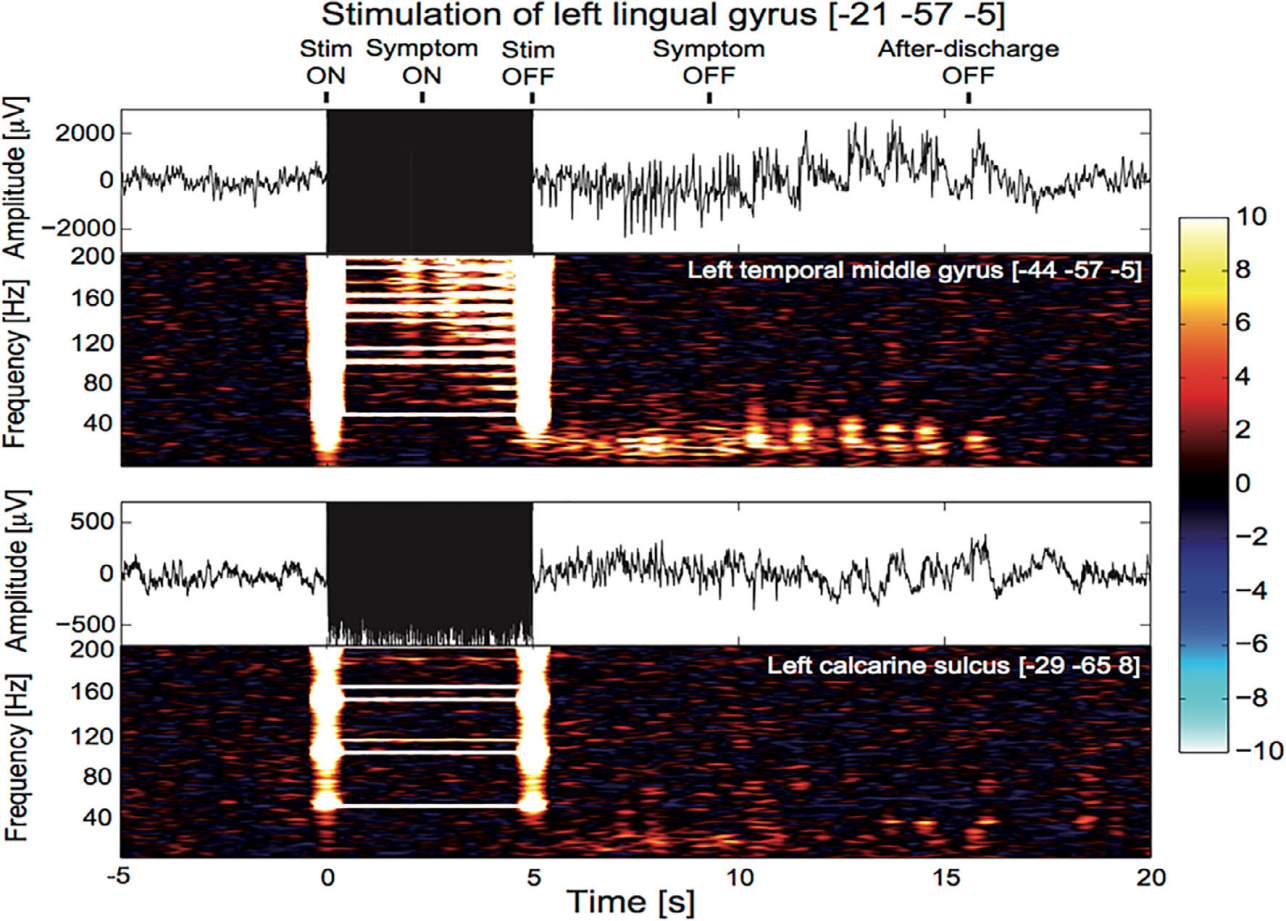
Example of stereo‐electroencephalographic (SEEG) recording obtained during 50 Hz DES of the left lingual gyrus (Montreal Neurological Institute [MNI] coordinates: [−21–57–5]). The stimulation was applied during 5 s (black rectangle liked artifacts) and recordings of the left middle temporal gyrus (top) and the left calcarine sulcus (bottom). Five events were positioned when reviewing the file: Start and end of the stimulation (Stim ON; Stim OFF; respectively), start and end of the language symptom (symptom ON; symptom OFF, respectively), and end of after‐discharge (after‐discharge OFF). Note that in this case, the symptom was reading speech arrest. The time–frequency decomposition of power of each channel (units: z‐score according to baseline levels) clearly shows the artifact of stimulation with vertical bars at the start and the end of stimulation and horizontal patterns at the frequency of stimulation and its harmonics. The left calcarine sulcus did not respond to the stimulation as no significant change of SEEG could be detected. In the left middle temporal gyrus, one can notice changes of SEEG power above 120 Hz in correlation with the clinical symptom (start between 2 and 3 s after stimulation onset). After the stimulation, the after‐discharge showed activity below 40 Hz

Each DES trial having induced a language interference was carefully reviewed in order to: (a) classify the language error (see above), allowing several errors to occur in the same trial and (b) add events to each SEEG files that will be used in further processing of SEEG data (see below). The events aimed at quoting (Figure 1):

- The start and end of the DES based on the stimulation artifact.
- The start and end of the language symptom based on the video.
- The end of the after‐discharge or fast oscillations induced by the stimulation, as such electrophysiological signature was visually identified in all instances.

#### 2.7.2 Processing of single SEEG trials

The processing of single SEEG trials was derived from the procedure we developed to map epileptogenicity, that is, fast oscillatory activity at seizure onset (David et al., 2011). In this framework, time series of spectral power in a given frequency band are first computed for each channel. At selected time bins, the spectral power is then log‐transformed and spatially interpolated to produce a series of images, which can then undergo statistical analyses in order to provide statistical parametric maps of SEEG power by comparing a period of interest to a baseline. Here, the baseline was a period of 10 s duration chosen with as little interictal epileptiform activity as possible, in the interval between 50 and 30 s before DES onset depending on the trials. The period of interest was of 3 s maximal duration, starting 800 ms after stimulation onset and stopping 800 ms before stimulation termination.

SEEG power in the time–frequency domain was obtained between 70 and 150 Hz using a Hanning‐tapered spectral decomposition with a time window of 1 s duration translated every 100 ms. The spectral resolution was chosen at 1 Hz and a notch filter was applied between 148 and 152 Hz to remove the third harmonic of line noise. Because DES produced an artifact on SEEG power (Figure 1), the next step of the analysis was to remove any effect of the stimulation artifact in inferred changes of SEEG power. To that aim, we chose to mask out the artifact in the time–frequency domain by ignoring aberrant values of SEEG power, which were assumed to be above 10 after z‐scoring power transform during DES by the transform during the baseline. All corresponding elements of the SEEG power matrix were thus ignored when computing SEEG power time series at every time bin by averaging values along the frequency dimension. In addition, as a conservative measure, SEEG power matrix elements with a z‐value above 10 in at least 5% of channels were also removed from all channels, that is even in channels where the value did not reach the threshold. The same processing steps were applied to the baseline data, even if there were no artifact to avoid any analysis bias in the final statistical comparison.

For mapping after‐discharges, similar analyses as for DES‐induced HFA were performed but the frequency band was set to 3–45 Hz, and the period of interest was set from the end of the stimulation period (*t* = 5 s) up to the termination of the after‐discharge.

Knowing SEEG electrode positions in a standardized coordinate system (MNI coordinates) and SEEG power values for each electrode, it is possible to create images of SEEG power by local spatial interpolation (David et al., 2011). We used an interpolation procedure that constrains voxels to the neocortical mantle, the hippocampus, and the amygdala, which are the structures the most frequently explored for epilepsy surgery in temporal lobe epilepsy patients. It works as follows (David, 2019): (a) for each electrode contact, the mesh vertices at a distance less than 1 cm are detected and (ii) to each vertex detected in the vicinity of at least one electrode, the assigned value is the average of the SEEG power values of the close electrodes, weighted by the inverse of the distance between the vertex and the electrodes (stronger weight is given to the closest electrodes). Once created, the images of SEEG log power were smoothed using an isotropic Gaussian kernel with a width of 3 mm (equivalent to the distance between successive SEEG electrodes) in order to control family‐wise error in the context of spatially correlated imaging data using the theory of Gaussian random fields (Worsley, Taylor, Tomaiuolo, & Lerch, 2004). Statistical significance of the difference between baseline and symptom periods (two sample *t*‐test) was directly obtained by the family‐wise error‐corrected associated *p*‐values. By applying a threshold on this *p*‐value (.05 corrected for multiple comparison), it was possible to determine the regions showing significant fast discharges concomitantly to the language symptoms during DES, for each trial.

#### 2.7.3 Group analysis

Group analysis of SEEG responses was performed for deriving maps of the probability of recording significant responses for each type of language error. We pooled together patients and events in conjunction analysis with the following steps:

1. For each trial, a binary map showing voxels having a significant SEEG power during the language error was obtained by thresholding the statistical maps of SEEG log power to *p* < .05.
2. Each surviving voxel was assigned to a specific ROI using a pre‐defined parcellation scheme. Every parcel with at least one activated voxel was considered as active, and thus a binary map of active (1) and nonactive (0) parcels was produced for each event. For this study, we used the AAL scheme (Tzourio‐Mazoyer et al., 2002).
3. The *group probability map of symptom‐related SEEG power* was obtained by averaging across trials the binary maps at ROI level. For each type of symptoms, we considered only ROI recorded at least 10 times to ensure a certain level of reproducibility in the results.

The values of the group probability map ranged between 1 (ROI systematically showing significant error‐related increase of HFA power when recorded) and 0 (ROI found to never show any significant symptom‐related increase of HFA power when recorded). We called those maps “HFA maps.”

A similar methodology was applied in order to produce maps of the probability to induce a particular symptom with DES for each ROI (number of times a symptom was induced when stimulating ROI *n* divided by the number of times ROI *n* was stimulated). We call those maps “DES maps.” For display purposes, those HFA and DES maps are shown in figures on a canonical inflated brain.

Finally, we used HFA maps to evaluate probabilistic functional cooccurrence of HFA elicited during naming symptoms on a language network composed of five ROIs. These five ROIs were significantly stimulated and recorded in our dataset: inferior frontal gyrus at the pars *triangularis* and *opercularis*; insula; superior and middle temporal gyrus. For that purpose, we simply computed the group probability using conjunction analysis, which gave a value between 1 (recorded ROIs systematically showing increased HFA power when one specific language‐related ROI presented a symptom during DES, independently of the symptom type) and 0 (recorded ROIs found to never show any HFA power modification when one specific language‐related ROI presented a language symptom during DES, independently to the symptom type). This ROI‐based analysis was duplicated to map the propagation of after‐discharges.

## 3 RESULTS

### 3.1 Visual analysis of SEEG‐video recordings

Visual analysis of DES responses as illustrated in Figure 1 indicated a median symptom onset of 1 s, with a median duration of 6.26 s. The median value of the termination of the observed postdischarge was 11.7 s. Temporal precision of behavioral measurements was, however, limited because of the difficulty to assess the dynamics of language symptoms from SEEG‐video recordings. One should also note that because of the huge stimulation artifact, it was not possible to visually measure the onset of postdischarge from SEEG recordings.

### 3.2 DES and naming errors

**FIGURE 2:**
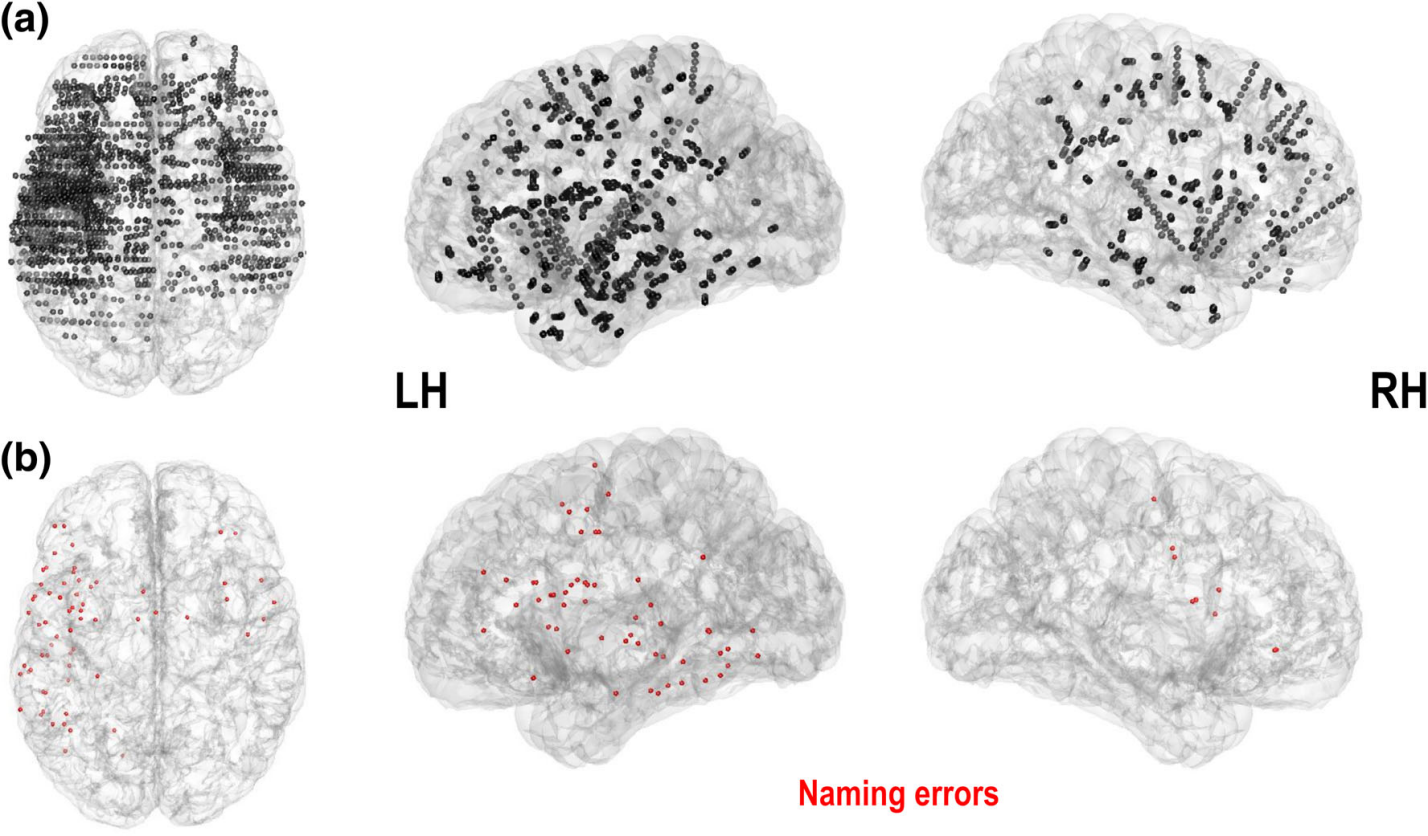
(a) Anatomical location of all cortical sites recorded during stereo‐electroencephalographic (SEEG). (b) Anatomical location of all cortical sites responsible for naming errors during direct electrical stimulation (DES) (in red). All data (P1–P29) were put together and represented onto a MNI brain template in the top and lateral (left and right) view

Figure 2 shows in an MNI template recorded electrodes localization in all patients (total of 2080 sites; 1,481 left‐sided and 599 right‐sided) and the electrodes localization in which DES induced naming interferences (total of 65 sites; 56 left‐sided and 9 right‐sided). A total of 104 naming interference could be associated with stimulation in these cortical sites (Table 2). Cortical sites were grouped in 23 ROI according to AAL parcellation (Tzourio‐Mazoyer et al., 2002), with 17 ROI in the left hemisphere and 6 in the right hemisphere. According to the raw data of naming errors (Table 2), the most frequently observed errors were naming SA (79.81%), naming PP (10.58%) followed by naming SP (9.62%). Naming errors were more frequently observed in the left dominant (LH, 87.50%) than in the right nondominant hemisphere (RH, 12.50%). Importantly, only NSA errors were observed in the right hemisphere.

**TABLE 2:** Naming error data by stimulated ROIs, including the hemisphere, the AAL label of each ROI, and the number of recorded errors in each ROI

H	AAL label	SA	SP	PP	Total
L	Frontal_Inf_Oper	8	0	1	9
	Frontal_Inf_Tri	5	0	0	5
	Frontal_Mid	0	0	3	3
	Fusiform	2	0	0	2
	Heschl	1	0	0	1
	Hippocampus	1	0	1	2
	Insula	7	2	0	9
	Lingual	2	1	0	3
	Postcentral	3	0	0	3
	Precentral	7	0	0	7
	Rolandic_Oper	2	0	0	2
	Supp_Motor_Area	6	0	0	6
	SupraMarginal	2	0	0	2
	Temporal_Inf	9	3	2	14
	Temporal_Mid	10	3	1	14
	Temporal_Pole_Sup	2	0	0	2
	Temporal_Sup	3	1	3	7
R	Cingulum_Mid	1	0	0	1
	Frontal_Inf_Oper	3	0	0	3
	Frontal_Inf_Orb	2	0	0	2
	Insula	2	0	0	2
	Precentral	2	0	0	2
	Rolandic_Oper	3	0	0	3
**Total**		83	10	11	104
**Percent**		79.81	9.62	10.58	

Abbreviations: AAL, automated anatomical labeling; H, hemisphere; LH, left hemisphere; RH, right hemisphere; PP, phonemic paraphasia; ROI, region of interest; SA, speech arrest; SP, semantic paraphasia.

### 3.3 DES and probabilistic naming error analysis

**FIGURE 3:**
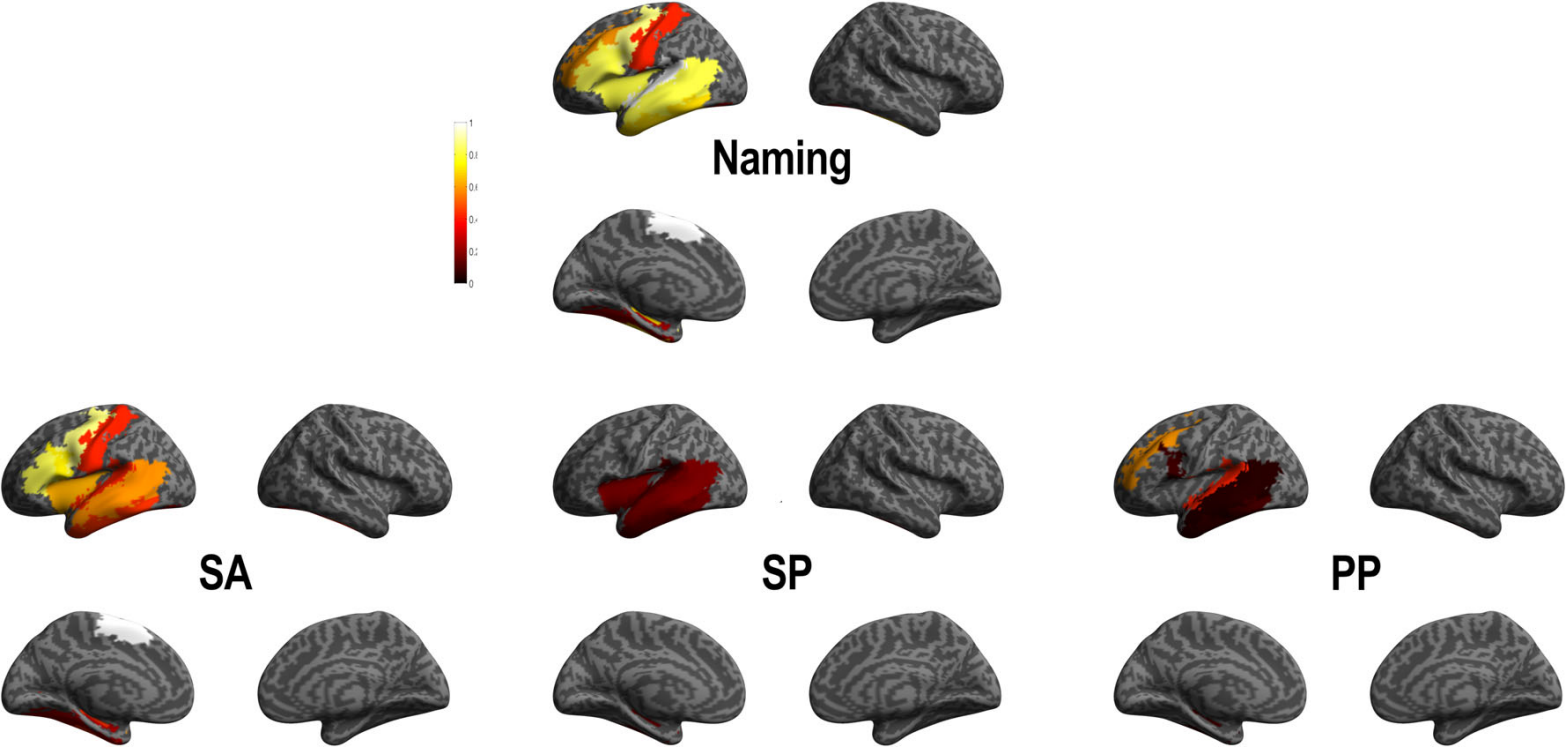
Direct electrical stimulation (DES) group probability map according to each type of naming errors. Lateral and medial views of the left and right hemisphere of Montreal Neurological Institute (MNI) brain template are represented. PP, phonemic paraphasia; SA, speech arrest; SP, semantic paraphasia. The color bar indicates the probability value (1: cortical stimulation systematically induced a naming errors in this region of interest (ROI); 0: cortical stimulation never induced a naming errors in this ROI)

To quantify the involvement of an ROI during naming errors we first reported the probability to observe a naming error, when stimulating the ROI (Table 3 and Figure 3). This probability was defined as the number of errors observed in one ROI over the total amount of stimulation with language interference performed in that same region. Only ROIs with at least three induced language interference were considered, which represented 11 ROIs of the left hemisphere (Table 3). A larger number of ROIs were related to the SA errors during DES than with the PP and with the SP. Specifically, SA errors were associated with DES performed in: left frontal regions (including the precentral gyrus, the interior frontal gyrus [IFG] on the pars triangularis and pars opercularis, the supplementary motor area [SMA], and the insula); left temporal regions (including the inferior, middle, and superior gyri as well as the fusiform gyrus) and left postcentral gyrus. SP errors were associated with DES performed on the left frontal (insula) and left temporal (including the inferior, middle, and superior temporal gyri) regions. While PP errors were associated with DES performed on the left frontal (at the level of the IFG in the pars opercularis and the middle frontal gyrus) and the left temporal (at the level of the inferior, middle, and superior gyri) cortex.

**TABLE 3:** Cortical regions (ROI, AAL labels) inducing language errors when stimulated and the related probability to observe a type of naming error

H	ROI (AAL)	SA	SP	PP	Total naming
LH	Precentral	.875	0	0	.875
	Frontal_Mid	0	0	.600	.600
	Frontal_Inf_Oper	.727	0	.091	.818
	Frontal_Inf_Tri	.833	0	0	.833
	Supp_Motor_Area	1	0	0	1
	Insula	.636	.181	0	.818
	Postcentral	.428	0	0	.428
	Fusiform	.250	0	0	.250
	Temporal_Sup	.428	.142	.428	1
	Temporal_Mid	.588	.176	.058	.823
	Temporal_Inf	.449	.150	.100	.699

Abbreviations: AAL, automated anatomical labeling; H, hemisphere; LH, left hemisphere; RH, right hemisphere; PP, phonemic paraphasia; ROI, region of interest; SA, speech arrest; SP, semantic paraphasia.

### 3.4 SEEG HFA and naming errors

**FIGURE 4:**
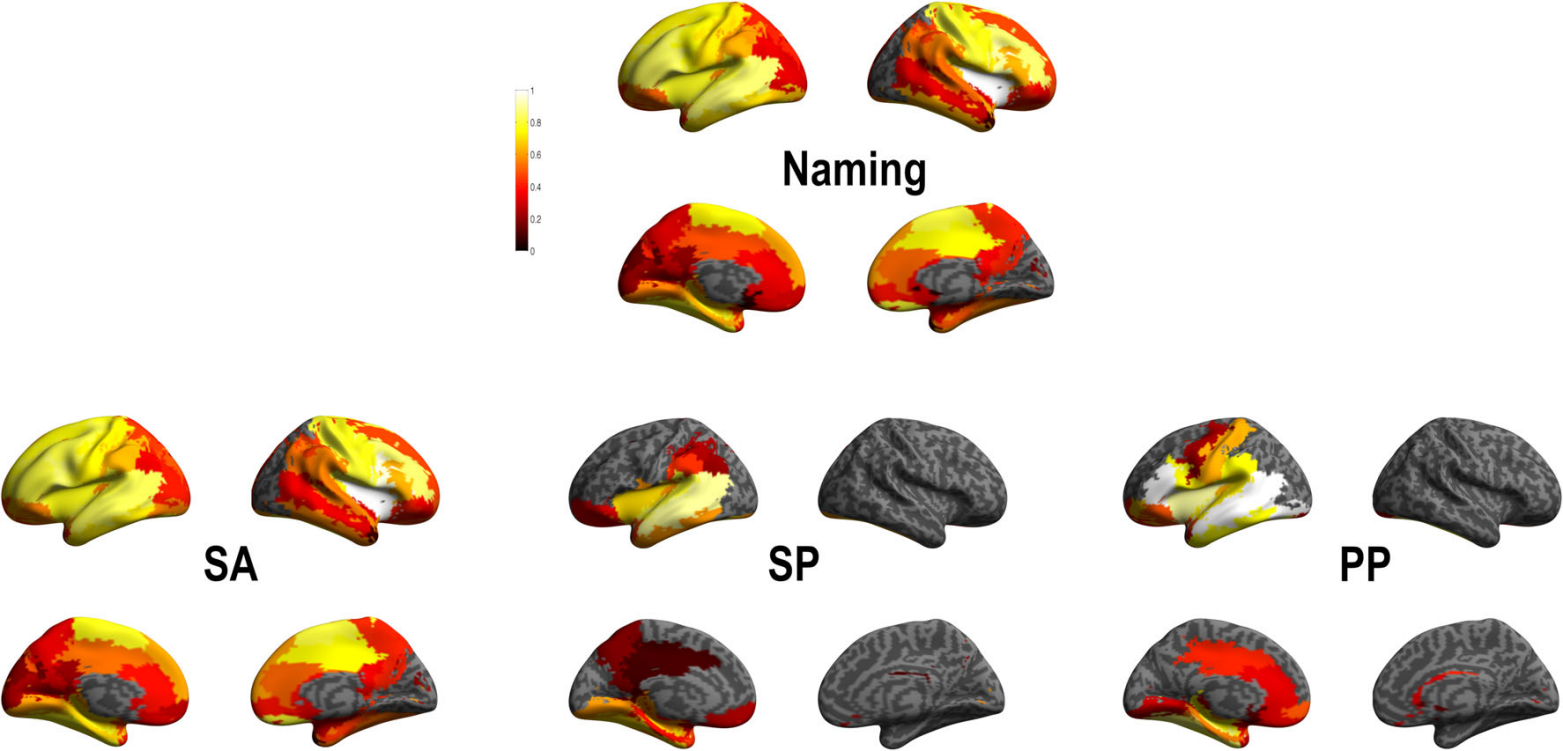
High frequency activity (HFA) group probability map according to each type of naming errors. Abbreviations same as in Figure 3. The color bar indicates the region of interest (ROI)‐based probability of recording HFA (1: cortical stimulation systematically induced a naming symptom‐related HFA in this ROI; 0: cortical stimulation never induced a naming symptom‐related HFA in this ROI)

We present now the SEEG HFA power modification observed during naming errors. We evaluated the significant HFA difference (*t* test) between: before and during induced DES naming errors (see Section 2). Figure 4 and Supplementary Table S1 summarize these results and show that regions with stimulation‐induced HFA were more extended than regions targeted by DES. The more extended anatomical coverage during DES induced‐language errors in terms of activity suggests that effective naming achievement was supported by a large language network. Furthermore, to be able to evaluate the relevance of HFA (70–150 Hz) for functional mapping, we performed the same analysis in other frequency bands (low gamma [30–70] Hz, and a broad low frequency band [2–25] Hz; see supplementary Figure S1 and supplementary Tables S3–S6 for such results). In brief, HFA showed higher probability values, thereby confirming its good correlative value for mapping brain functions.

Specifically, a large bilateral cerebral network was observed during SA errors including the left and right frontal, parietal, and temporal cortices and left occipital regions. SP errors involved a left lateralized network including similar regions to those involved during SA errors but less extended and more concentrated at the level of the temporal lobe. The left cerebral network involved during PP errors included a fronto‐parieto‐temporal network included in the SA network and more extended that the network observed for SP (see supplementary Table S1 for details).

### 3.5 SEEG and naming subnetworks

**FIGURE 5:**
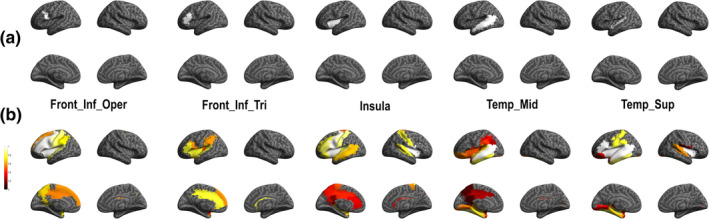
High frequency activity (HFA) group probability map for each region of interest (ROI). Top: anatomical location of ROI (seed) analysis and down: functional subnetwork results. The color bar indicates the ROI‐based probability of recording HFA (1: cortical stimulation systematically induced a symptom‐related HFA increase in this ROI; 0: cortical stimulation never induced a symptom‐related HFA increase in this ROI)]

In order to evaluate brain language functional subnetworks related to naming, we evaluated HFA induced by DES in core language regions. Specifically, we designated five ROIs: the left inferior frontal gyrus at the pars opercularis and pars triangularis, the left insula, the left middle temporal and superior temporal cortex. This ROI analysis allowed us to evaluate the probability to observe an HFA change in a near or in a far region from these core language regions stimulation and reveal functional subnetworks (Figure 5 and Supplementary Table S2 as well as supplementary Figure S2 and Table S7 for results in other frequency bands).

We showed that stimulation of the left inferior frontal gyrus at the pars opercularis ROI was related with HFA modification in a large left fronto‐parieto‐temporal network (see supplementary Table S2 for all probability value). The strongest probabilities (*p* = 1) showed in relation with this ROI were observed in six left lateralized frontal regions (including: inferior gyrus at the level of the pars opercularis and pars triangularis, Rolandic operculum, precentral gyrus, insular cortex, and middle frontal cortex). Stimulation of the left inferior frontal gyrus at the pars triangularis ROI also induced HFA modification in a fronto‐parieto‐temporal network, but with a more restricted number of brain regions compared with the pars opercularis ROI. Indeed, strongest probabilities (*p* > .8 here; no region with *p* = 1 was observed) were showed in five left cortical regions (including inferior frontal gyrus in the pars triangularis, insula, temporal superior gyrus, anterior, and middle cingulum). Stimulation of the left insula ROI showed HFA modification in an extended bilateral fronto‐parieto‐temporal network, in which the strongest probabilities (*p* = 1) were observed in left and right frontal (including: left and right Rolandic operculum, left and right insula, left pars opercularis, and precentral gyrus). The middle temporal gyrus ROI was related to response patterns in fronto‐parieto‐temporal network and the strongest probability (*p* = 1) is only observed in this same region. Finally, stimulation of the superior temporal gyrus ROI showed HFA modification in an extended bilateral fronto‐parieto‐temporal network, in which strongest probabilities were observed in eight regions including left and right frontal (left inferior frontal gyrus at the pars opercularis and triangularis, Rolandic operculum, left and right insula) and left temporal (middle and superior temporal cortices, as well as the Hesch'l gyrus) regions.

**FIGURE 6:**
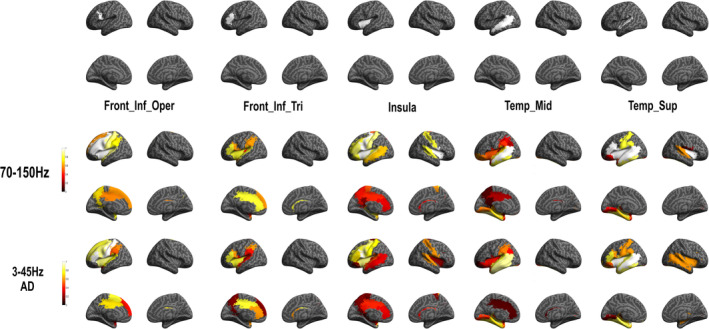
Stereo‐electroencephalographic (SEEG) group probability map for each region of interest (ROI) for high frequency activity (HFA) (70–150) Hz and (3–45) Hz (after‐discharge [AD]). Top: anatomical location of ROI (seed) analysis and down: functional subnetwork results for each frequency band. See for probability values supplementary Table S7

Interestingly, the ROI analysis of HFA responses and of after‐discharges showed very similar patterns, although the probability of HFA responses was found slightly higher (Figure 6 and supplementary Table S7 for probability values). It demonstrates that the areas connected to the stimulated site can be inferred from both types of features.

## 4 DISCUSSION

In the present study, we bring new evidence supporting the view that language is the result of distributed populations of interconnected and synchronized neurons, including subnetworks rather than to isolate functional areas, related to different language processes (Duffau et al., 2008; Duffau et al., 2014). Our hypothesis was that cortical sites related to language disturbances (naming errors) during DES, may be considered as input gates into a larger language network (Mandonnet et al., 2010). We identified and put in relation both cerebral regions related to DES‐induced‐naming errors and language networks based on HFA (70–150 Hz) modifications, by using a specific probabilistic methodological approach combining DES and SEEG recordings.

DES raw data indicated that naming errors were more frequently observed in the left hemisphere (79.81%), in agreement with the left hemisphere specialization for language processing (Tzourio‐Mazoyer, Perrone‐Bertolotti, Jobard, Mazoyer, & Baciu, 2017). This result is also corroborated by DES probability analysis showing a restricted left lateralized fronto‐temporo‐parietal network (Table 3) during naming errors. It is important to note that only one patient included in the present study presented a right hemispheric predominance for language as assessed by fMRI, therefore, its SEEG implantation was in the left nonspecialized hemisphere (Table 1). Furthermore, the seven patients with right hemisphere SEEG implantation presented left hemispheric specialization for language. Thus, the right hemisphere sampling in the present study is representative of a right nonspecialized hemisphere.

Raw data also indicated that SA was the most prevalent naming error type followed by phonemic paraphasia (PP), and the less prevalent was SP (Table 2). In line with these results, DES probability group analysis showed that SA was induced by DES performed in a larger perisylvian network than PP and then SP. Important PP and SP were shown in DES performed in similar brain regions than SA, and only PP errors were observed on a supplementary region, outside the common network, the middle frontal cortex. This result suggests a common language network for the three types of naming errors evaluated in the present study (Table 3). It is important to note that in the present study, we were unable to differentiate the SA symptom induced by motor difficulties or by lexico‐semantic access (i.e., anomia). Indeed, Mandonnet (2017) suggested that to be able to made this difference during picture naming, the patient should always be asked to say aloud automatic sentences “This is a….” Unfortunately, this methodology was not used in the present study, which is a retrospective analysis of data recorded in a clinical routine setup.

The common network observed in the present study during DES for the three types of language symptoms could be explained by the low rate of PP and SP types of errors (~9 and 10% of the total number of observed errors, respectively), similarly to previous DES findings (Corina et al., 2010; Duffau et al., 2014). It is possible that SA errors observed during perisylvian areas stimulation induces a complete inhibition of language output and preventing to verify whether PP or SP processing has been distributed. In line with this assumption, in the present study, all the DES cortical regions inducing SA covert the DES cortical regions inducing PP and SP.

Specifically, PP were observed during DES performed on lateral middle frontal, inferior frontal (*pars opercularis*) and lateral temporal cortices; SP errors were observed during DES performed on insula and lateral temporal cortices (Table 3). PP and SP cortical distribution can be related to the dual stream organization for naming (Duffau et al., 2014), including a ventral (fronto‐temporal) and a dorsal (fronto‐temporo‐parietal) pathway. The ventral stream supports processing of information from visual analysis to meaning, reflected in our study by SP and SA errors induced by the DES on temporal cortices. The dorsal stream is rather involved in processing of information from visual analysis to overt articulation; these processes are reflected in our study by PP and SA errors induced by DES applied on fronto‐temporal and insular cortices (Table 3 and Figure 3).

The dual stream organization during naming was supported by the SEEG HFA analysis (Table S1 and Figure 4) which highlighted the massive coactivation observed at intrahemispheric and interhemispheric levels. The DES‐induced SA errors were associated with high probability of HFA changes in an extended coactivated bilateral fronto‐parieto‐temporal network including the left occipital gyrus (related to visual processing of pictures, see Table S1). The probability of HFA changes induced by DES associated with PP and SP errors, concerned a left restricted and less extended network (Table S1).

We were also able, by the analysis of ROI related to HFA coactivation or to after‐discharges, to identify different language subnetworks. We put in relation these subnetworks with literature results on naming. Specifically, at least four linguistic processes underlying picture naming could be proposed: visual perception, semantic retrieval, phonological representation, and speech articulation (Duffau et al., 2014; Indefrey & Levelt, 2004; Lau et al., 2015; Rofes et al., 2018). Our ROI analysis showed that the inferior frontal gyrus (*pars opercularis*) is related with an extended left lateralized network including cortical regions identified as related with the three of the four linguistic processing involved on naming. Specifically, coactivated regions including medial temporal cortices and the temporal pole (semantic retrieval), phonological representations (fronto‐parietal regions) and motor, premotor and inferior frontal regions (motor planning and speech articulation). A recent study revisiting the brain subnetwork involved during naming process identified the inferior frontal gyrus (*pars opercularis*) as core region on the phonological, syntactic and cognitive control hub supported by the extended anatomical connectivity with frontal, temporal and prefrontal regions (Duffau et al., 2014). Furthermore, the involvement of the inferior frontal gyrus can be related to the early (executive and top‐down predictions) or late (phonological‐articulatory processes) dynamics previously observed during language processing (Perrone‐Bertolotti et al., 2020; Yvert, Perrone‐Bertolotti, Baciu, & David, 2012) and specifically during naming (Miozzo, Pulvermüller, & Hauk, 2015; Popescu et al., 2017).

Results for the left inferior frontal gyrus pars triangularis, revealed coactivation within a restricted left lateralized frontal network including regions implicated in planning, motor execution as well as phonological and semantic processing. Interestingly an important involvement of the anterior cingulate cortex/medial superior frontal cortex (dACC/msFC) was found. The ACC/msFC which is known to be a key region of the cingulo‐opercular network, which is one of the two top‐down (control) networks in human and is specifically involved in set maintenance and in monitoring (making choices) in accordance with the task goal [see Dosenbach, Fair, Cohen, Schlaggar, & Petersen, 2008]. Our findings are thus in line with several results suggesting an important implication of control semantic selection and regulatory mechanisms during naming (e.g., Schnur, Schwartz, Brecher, & Hodgson, 2006; Thompson‐Schill, Bedny, & Goldberg, 2005; Thompson‐Schill, D'Esposito, Aguirre, & Farah, 1997). In particular, during naming the production of the target word implies a selection from a competing set of other words, this selection mechanism implied regulatory cognitive processing by biasing competitive interaction among incompatible representations (Schnur et al., 2009). We also showed a more extended number of regions with less probability results, including parietal and medial temporal regions. Taken together, the IFG responses shown in the present study are in line with previous findings suggesting that the IFG may represent a supramodal hierarchical processor (Tate, Herbet, Moritz‐Gasser, Tate, & Duffau, 2014; Tettamanti & Weniger, 2006) involved in specific language information processing (Bookheimer, 2002; Perrone‐Bertolotti, Kauffmann, Pichat, Vidal, & Baciu, 2017) and in a cognitive control during language tasks.

The left mid temporal ROI is related to a restricted left fronto‐temporal network which shows the strongest probability of stimulation‐induced HFA responses (*p* = 1) for lateral temporal regions. Others regions with less probability were observed in the medial temporal cortex (hippocampus, parahippocampus, amygdala), lateral and medial parietal cortex (including the supramarginal, angular gyrus, precuneus, inferior parietal cortex). This result suggests a semantic subnetwork (Binder, Desai, Graves, & Conant, 2009), and in relation with supramodal integration and concept retrieval as proposed in previous studies focused on the left mid temporal cortex. Supported also by semantic deficits observed after damage of this region (Dronkers, Wilkins, van Valin, Redfern, & Jaeger, 2004; Hillis & Caramazza, 1991).

Only the insula and the superior temporal ROIs showed bilateral HFA network representation. Indeed, the left insula showed the strongest coactivation with left and right frontal regions, as well as right insula. We interpret this subnetwork as reflecting articulatory processes and executive control required by language production. Indeed, insula is involved in articulatory *planning* (Dronkers, 1996; Price, 2012), articulatory planning and control processes (Ackermann & Riecker, 2010; Lau et al., 2015) as well as in decision‐making (Droutman, Bechara, & Read, 2015). Finally, results for left superior temporal ROI showed extended bilateral fronto‐parieto‐temporal network including several occipital regions and possibly related to semantic and phonological integration during visual processing. According to its anatomical location, this ROI may ensure the interplay between the dorsal and ventral streams (Duffau et al., 2014; Moritz‐Gasser, Herbet, & Duffau, 2013; Vigneau et al., 2006). Indeed, DES findings showed that the superior temporal gyrus has a multimodal organization with neurons involved in both visual recognition and naming (Roux et al., 2015). Furthermore, the superior temporal cortex was involved in word retrieval during a verbal self‐monitoring task (Hocking, McMahon, & de Zubicaray, 2009; Price, 2010), which uses similar processes as picture naming performed by our patients.

In addition, we also explored medial temporal regions such as the hippocampus. Although the DES applied to this region did not induce language symptoms, the HFA SEEG recordings revealed significant HFA responses in the left hippocampus and bilateral parahippocampus during SA and SP errors. Interestingly, ROI analysis revealed that the hippocampus and the parahippocampal gyri were involved in three of five explored subnetworks (absent for the pars triangularis and pars opercularis ROI analysis, see Table S2). Medial temporal regions are generally less considered by the language models, although they can have a role during word production (Price, 2012), as suggested by neuropsychological data in patients with hippocampal sclerosis (Bonelli et al., 2011; Hamberger et al., 2007; Lau et al., 2015). Hamberger (2015) highlighted that the role of the hippocampus during language production and more specifically during naming. Recently, the hypothesis that the hippocampus plays an essential role on the association between the identity (picture) and the corresponding label (name) retrieval during naming was evaluated (Hamamé, Alario, Llorens, Liégeois‐Chauvel, & Trébuchon‐Da Fonseca, 2014). These authors recorded HFA (50–150 Hz) in SEEG during a naming task and showed that hippocampus activity latency predicted the naming behavioral latency. Most interestingly, they showed that the absence of the hippocampus activity was related to difficult naming (e.g., “tip‐of‐the‐tongue”). Our results support the idea that the hippocampus is involved during language production and more generally in a global process of incoming visual information and linguistic output retrieval association.

One major limitation of the present study is the limited number of patients included (*N* = 29). Although this cohort is large for an SEEG study, SEEG anatomical implantation is different between patients, as they depend on clinical assumptions. This implies that a larger number of patients would have been needed to be able to have a good anatomical coverage of the full‐language network. The limited number of patients included in the study prompted us to conduct an analysis in terms of ROIs. This ROI analysis is also a major limitation of our study because we decreased the inherent anatomical precision of SEEG. Another limitation of the present study is related to the more important left hemisphere sampling, including 1,481 sites on the left and 599 sites in the right hemisphere. This suggests that even if the majority of the included patients (28/29) presented a left hemisphere specialization for language, the results related to the hemispheric involvement during naming should be taken with caution due to the sampling bias in favor of the left hemisphere. Finally, to have better insight into the specificity of induced fast oscillations for language function, it would have been extremely relevant to contrast the responses to the same stimulated sites with versus without induced symptoms. However, asymptomatic electrophysiological DES data was not available in our clinical setup and we could not proceed to this type of analysis.

In conclusion, our study supports the idea that DES‐induced language dysfunction could be more the results of the modulation of large‐scale networks than the perturbation of one single cortical region, explaining that similar symptoms could be shown in different and distant cortical regions. Our results highlighted the feasibility of a functional mapping approach based on the combination DES–SEEG methods and suggest that HFA (70–150 Hz) unveil brain regions connected to the stimulated site with a better specificity than other frequency band. These results may open a new window onto the assessment of functional connectivity as a reflect of functional integrity of a cognitive subnetworks in a clinical context.

## CONFLICT OF INTEREST

The authors declare no conflicts of interest.

## ETHICS STATEMENT

Patients provided written informed consent to participate in the study, which was approved by the local ethics committee.

## Supporting information


**Appendix** **S1** Supporting Information

## Data Availability

The data that support the findings of this study are available from the Grenoble Hospital Center (CHUGA). Restrictions apply to the availability of these data, which were used under license for this study. Data are available from the authors with the permission of the CHUGA.
