# Supplementary material for: Probabilistic mapping of language networks from high frequency activity induced by direct electrical stimulation
Source: Hum Brain Mapp. 2020 Jul 22;41(14):4113–26. doi: 10.1002/hbm.25112 (PMC7469846; doi:10.1002/hbm.25112)
Supplement: Supplementary file 1 — Appendix S1 Supporting Information [file HBM-41-4113-s001.docx]

**Probabilistic mapping of language networks from high frequency activity induced by direct electrical stimulation**

**Supplementary materials**

Marcela Perrone-Bertolotti ^a.b+*^. Sarah Alexandre ^c+^. Anne-Sophie Job b ^c.d.e^. Luca De Palma^c^. Monica Baciu ^a.b^ . Marie-Pierre Mairesse ^c^. Dominique Hoffmann ^f^. Lorella Minotti ^c.d.e^. Philippe Kahane ^c.d.e^. Olivier David ^d.e^

^a^ Univ. Grenoble Alpes. Univ. Savoie Mont Blanc. CNRS. LPNC. 38000 Grenoble. France

^b^ Institut Universitaire de France

^c^ CHU Grenoble Alpes. Pôle Neurologie Psychiatrie. F-3800. Grenoble. France

^d^Univ. Grenoble Alpes. Grenoble Institut Neurosciences. GIN. F-38000 Grenoble. France

^e^ Inserm. U1216. F-3800. Grenoble. France

^f^ CHU Grenoble Alpes. Pôle Tête et Cou. F-3800. Grenoble. France

+ Both authors contributed equally to this work

*** Corresponding author:**

Marcela Perrone-Bertolotti. PhD

Laboratoire de Psychologie et Neurocognition

UFR Sciences de l’Homme et de la Société

CS 40700

38058 Grenoble Cedex 9

mail: [marcela.perrone-bertolotti@univ-grenoble-alpes.fr](mailto:marcela.perrone-bertolotti@univ-grenoble-alpes.fr)

Tel: +33476825860

**Supplementary Table 1**

|  |  | **LEFT HEMISPHERE** | | | |  | **RIGHT HEMISPHERE** | | | |
| --- | --- | --- | --- | --- | --- | --- | --- | --- | --- | --- |
| **LOBE** | **ROI (AAL)** | **SA** | **SP** | **PP** | **Naming** |  | **SA** | **SP** | **PP** | **Naming** |
| **Frontal** | Frontal_Inf_Oper | **.860** |  | **.750** | **.850** | Insula | **1** |  |  | **1** |
|  | Precentral | **.818** |  | **.250** | **.754** | Frontal_Inf_Oper | **.962** |  |  | **.900** |
|  | Frontal_Mid | **.813** |  |  | **.833** | Frontal_Mid | **.857** |  |  | **.857** |
|  | Frontal_Inf_Tri | **.811** |  | **1** | **.841** | Supp_Motor_Area | **.810** |  |  | **.810** |
|  | Supp_Motor_Area | **.783** |  |  | **.760** | Rolandic_Oper | **.808** |  |  | **.710** |
|  | Rolandic_Oper | **.778** | **.600** | **.727** | **.753** | Rectus | **.800** |  |  | **.800** |
|  | Insula | **.773** | **.700** | **.909** | **.781** | Precentral | **.773** |  |  | **.773** |
|  | Frontal_Sup | **.756** |  |  | **.739** | Cingulum_Mid | **.750** |  |  | **.750** |
|  | Frontal_Mid_Orb | **.643** | .375 | **.667** | **.595** | Frontal_Inf_Tri | **.636** |  |  | **.636** |
|  | Frontal_Sup_Medial | **.620** |  |  | **.618** | Frontal_Sup_Medial | **.611** |  |  | **.611** |
|  | Frontal_Inf_Orb | **.571** | .333 | .500 | .525 | Cingulum_Ant | .524 |  |  | .524 |
|  | Cingulum_Mid | .543 | .111 | .429 | .488 | Frontal_Sup_Orb | .500 |  |  | .500 |
|  | Frontal_Sup_Orb | .486 | .375 | .400 | .453 | Frontal_Sup | .476 |  |  | .476 |
|  | Cingulum_Ant | .397 | 0 | .333 | .351 | Frontal_Inf_Orb | .417 |  |  | .417 |
|  | Rectus | .321 | .250 | .333 | .310 | Frontal_Med_Orb | .400 |  |  | .400 |
|  | Frontal_Med_Orb | .280 | .250 | .500 | .308 | Frontal_Mid_Orb | .375 |  |  | .375 |
| **Temporal** | Temporal_Mid | **.836** | **.900** | **1** | **.863** | Heschl | **.714** |  |  | .273 |
|  | Temporal_Sup | **.803** | **.800** | **.727** | **.793** | Temporal_Pole_Sup | **.588** |  |  | .533 |
|  | Fusiform | **.778** | **.700** | **.857** | **.774** | Temporal_Inf | **.571** |  |  | .320 |
|  | Temporal_Inf | **.723** | **.600** | **.750** | **.708** | Temporal_Sup | **.560** |  |  | .333 |
|  | Hippocampus | **.691** | **.600** | **.750** | **.685** | Fusiform | .545 |  |  | **.654** |
|  | ParaHippocampal | **.689** | .400 | **.571** | **.629** | ParaHippocampal | .455 |  |  | **.625** |
|  | Temporal_Pole_Sup | **.638** | .500 | .500 | **.605** | Temporal_Mid | .350 |  |  | .111 |
|  | Heschl | **.569** | .600 | .636 | **.581** | Amygdala | .300 |  |  | **.583** |
|  | Amygdala | .475 | .300 | .375 | .442 | Hippocampus | .294 |  |  | .500 |
|  | Temporal_Pole_Mid | .419 | .444 | .286 | .404 | Temporal_Pole_Mid | .111 |  |  | **.619** |
| **Parietal** | Paracentralobule | **.771** |  |  | **.737** | Postcentral | **.800** |  |  | **.808** |
|  | Postcentral | **.733** |  | **.625** | **.704** | SupraMarginal | **.571** |  |  | **.571** |
|  | Parietal_Inf | **.642** | .250 |  | **.594** | Angular | .545 |  |  | .545 |
|  | SupraMarginal | **.641** | .444 | **.750** | **.630** | Parietal_Inf | .462 |  |  | .462 |
|  | Parietal_Sup | .462 |  |  | .429 | Paracentral_Lobule | .440 |  |  | .440 |
|  | Precuneus | .359 | .200 |  | .333 | Precuneus | .429 |  |  | .429 |
|  | Angular | .351 | .222 |  | .340 | Cingulum_Post | .364 |  |  | .364 |
|  | Cingulum_Post | .250 | .111 |  | .230 |  |  |  |  |  |
|  | Cuneus | .188 |  |  | .150 |  |  |  |  |  |
| **Occipital** | Lingual | **.611** | **.600** | .286 | **.566** |  |  |  |  |  |
|  | Occipital_Inf | **.571** |  | **1** | **.667** |  |  |  |  |  |
|  | Occipital_Sup | .500 |  |  | .429 |  |  |  |  |  |
|  | Occipital_Mid | .417 |  |  | .333 |  |  |  |  |  |
|  | Calcarine | .250 |  |  | .222 |  |  |  |  |  |

***Supplementary Table 1:*** *SEEG group probability values during each type of language error according to AAL labels.* In bold are highlighted the higher probability values (P> .55) observed according to cortical regions in each hemisphere and each lobe. *Abbreviation:* Speech Arrest (SA); Semantic Paraphasia (SP); Phonemic Paraphasia (PP).

**Supplementary Table 2**

| **Frontal_Inf_Oper** | | **Frontal_Inf_Tri** | | **Insula** | | **Temporal_Mid** | | **Temporal_Sup** | |
| --- | --- | --- | --- | --- | --- | --- | --- | --- | --- |
| **Rolandic_Oper_L** | **1** | Frontal_Inf_Tri_L | .800 | **Rolandic_Oper_L** | **1** | **Temporal_Mid_L** | **1** | **Rolandic_Oper_L** | **1** |
| **Frontal_Inf_Oper_L** | **1** | Insula_L | .800 | **Frontal_Inf_Oper_L** | **1** | Fusiform_L | .900 | **Frontal_Inf_Oper_L** | **1** |
| **Frontal_Inf_Tri_L** | **1** | Temporal_Sup_L | .800 | **Insula_L** | **1** | Temporal_Inf_L | .800 | **Frontal_Inf_Tri_L** | **1** |
| **Insula_L** | **1** | Cingulum_Mid_L | .800 | **Precentral_L** | **1** | Temporal_Sup_L | .700 | **Insula_L** | **1** |
| **Frontal_Mid_L** | **1** | Cingulum_Ant_L | .800 | **Rolandic_Oper_R** | **1** | Hippocampus_L | .700 | **Heschl_L** | **1** |
| **Precentral_L** | **1** | Rolandic_Oper_L | .600 | **Insula_R** | **1** | ParaHippocampal_L | .700 | **Temporal_Sup_L** | **1** |
| Heschl_L | .875 | Frontal_Inf_Oper_L | .600 | Temporal_Sup_L | .875 | Frontal_Mid_Orb_L | .625 | **Temporal_Mid_L** | **1** |
| Temporal_Sup_L | .875 | Temporal_Pole_Sup_L | .600 | Postcentral_L | .875 | Frontal_Inf_Orb_L | .556 | **Insula_R** | **1** |
| Postcentral_L | .875 | SupraMarginal_L | .600 | Frontal_Inf_Tri_L | .857 | Lingual_L | .556 | Hippocampus_L | .833 |
| Amygdala_L | .750 | Parietal_Inf_L | .600 | Hippocampus_L | .833 | Insula_L | .500 | Temporal_Inf_L | .833 |
| Temporal_Pole_Sup_L | .750 | Frontal_Sup_Medial_L | .600 | Frontal_Mid_L | .800 | Temporal_Pole_Sup_L | .500 | Fusiform_L | .833 |
| SupraMarginal_L | .750 | Heschl_L | .400 | Frontal_Inf_Orb_L | .800 | Frontal_Sup_Orb_L | .500 | Postcentral_L | .800 |
| Precuneus_L | .714 | Amygdala_L | .400 | Heschl_R | .800 | SupraMarginal_L | .444 | SupraMarginal_L | .800 |
| Parietal_Inf_L | .714 |  |  | Temporal_Sup_R | .800 | Rolandic_Oper_L | .400 | ParaHippocampal_L | .667 |
| Frontal_Sup_Medial_L | .600 |  |  | Postcentral_R | .800 | Parietal_Inf_L | .333 | Heschl_R | .600 |
| Frontal_Sup_L | .600 |  |  | Heschl_L | .750 | Temporal_Pole_Mid_L | .333 | Temporal_Sup_R | .600 |
| Supp_Motor_Area_L | .600 |  |  | Amygdala_L | .667 | Cingulum_Mid_L | .250 | Temporal_Pole_Sup_L | .500 |
| Cingulum_Mid_L | .571 |  |  | Temporal_Pole_Sup_L | .667 | Angular_L | .250 | Amygdala_L | .333 |
|  |  |  |  | Temporal_Mid_L | .667 | Amygdala_L | .222 | Frontal_Inf_Orb_L | .333 |
|  |  |  |  | Paracentral_Lobule_R | .600 | Heschl_L | .200 | Temporal_Pole_Mid_L | .333 |
|  |  |  |  | Paracentral_Lobule_L | .500 | Precuneus_L | .125 | Lingual_L | .333 |
|  |  |  |  | Cingulum_Mid_L | .429 | Cingulum_Post_L | .111 | Rolandic_Oper_R | .200 |
|  |  |  |  | Precuneus_L | .400 |  |  | Frontal_Sup_Orb_L | .167 |
|  |  |  |  | Cingulum_Ant_L | .333 |  |  |  |  |
|  |  |  |  | Cingulum_Post_L | .200 |  |  |  |  |

***Supplementary Table 2****: SEEG group probability values observed during the stimulation on each of the five languages ROI and according to AAL labels.* In grey are highlighted the higher probability values (P>.55) and in bold the regions with a P =1 *(1: cortical stimulation in one of the selected ROI systematically induces HFA in such AAL regions).*

**Supplementary Figure 1**

**
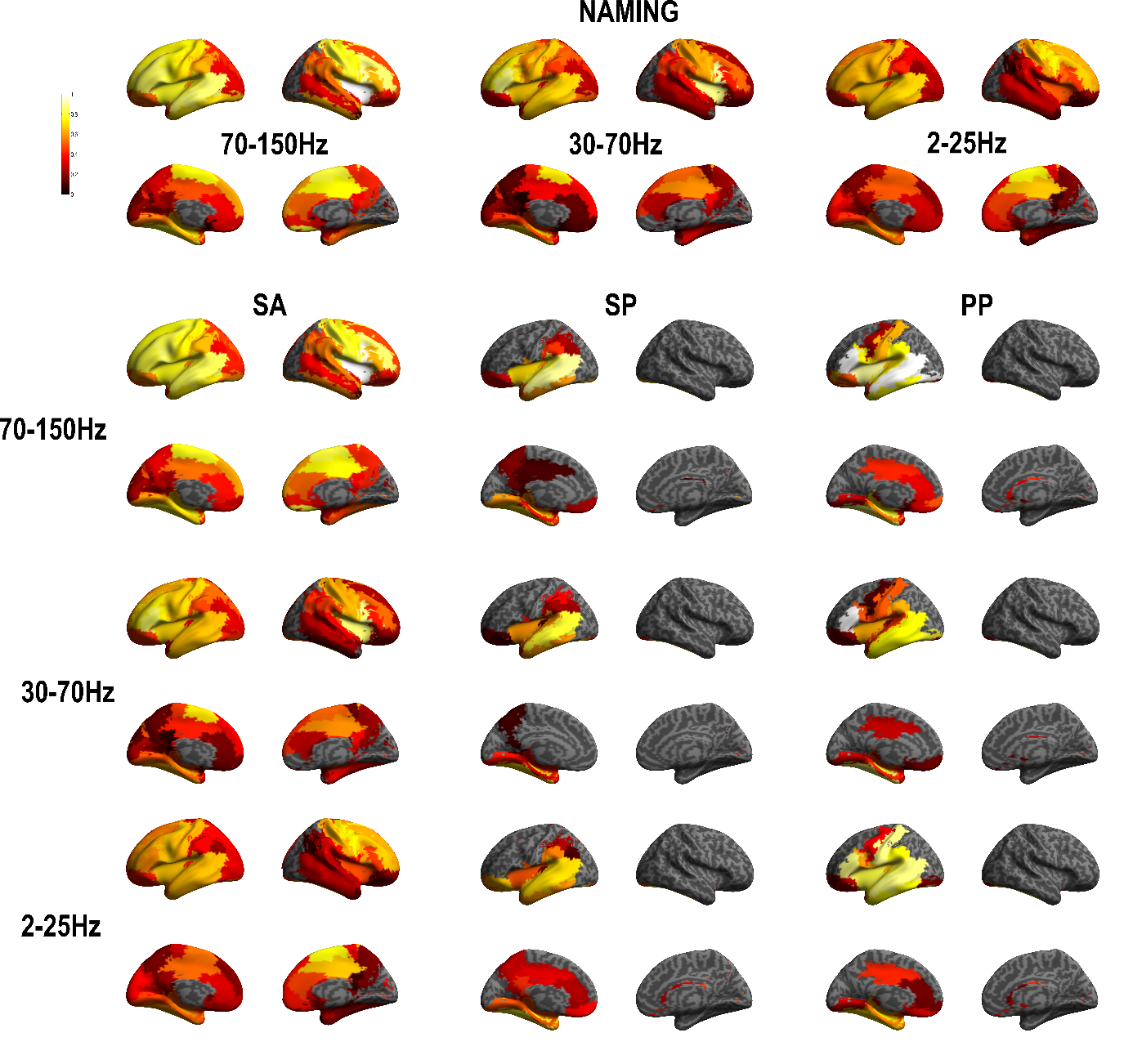
**

***Supplementary Figure 1:*** *SEEG group probability map for different frequency bands according to each type of explored language symptoms. Probability values are higher in 70-150Hz band than in the others bands.*

**Supplementary Table 3**

|  | **SPEECH ARREST** | | | | | | | |
| --- | --- | --- | --- | --- | --- | --- | --- | --- |
|  | **LH** | | | | **RH** | | | |
| **LOBE** | **ROI (AAL)** | **70-150 Hz** | **30-70 Hz** | **2-25 Hz** | **ROI (AAL)** | **70-150 Hz** | **30-70 Hz** | **2-25 Hz** |
| **Frontal** | Frontal_Inf_Oper | .860 | .740 | .660 | **Insula** | **10** | .862 | .483 |
|  | Precentral | .818 | .673 | .673 | Frontal_Inf_Oper | .962 | .885 | .615 |
|  | Frontal_Mid | .813 | .729 | .625 | Frontal_Mid | .857 | .524 | .667 |
|  | Frontal_Inf_Tri | .811 | .849 | .585 | Supp_Motor_Area | .810 | .524 | .762 |
|  | Supp_Motor_Area | .783 | .717 | .543 | Rolandic_Oper | .808 | .692 | .385 |
|  | Rolandic_Oper | .778 | .639 | .667 | Rectus | .800 | .000 | .400 |
|  | Insula | .773 | .693 | .667 | Precentral | .773 | .636 | .727 |
|  | Frontal_Sup | .756 | .634 | .561 | Cingulum_Mid | .750 | .583 | .667 |
|  | Frontal_Mid_Orb | .643 | .160 | .250 | Frontal_Inf_Tri | .636 | .409 | .455 |
|  | Frontal_Sup_Medial | .620 | .360 | .389 | Frontal_Sup_Medial | .611 | .444 | .200 |
|  | Frontal_Inf_Orb | .571 | .452 | .405 | Cingulum_Ant | .524 | .286 | .476 |
|  | Cingulum_Mid | .543 | .414 | .529 | Frontal_Sup_Orb | .500 | .250 | .343 |
|  | Frontal_Sup_Orb | .486 | .429 | .250 | Frontal_Sup | .476 | .238 | .619 |
|  | Cingulum_Ant | .397 | .175 | .317 | Frontal_Inf_Orb | .417 | .250 | .167 |
|  | Rectus | .321 | .143 | .214 | Frontal_Med_Orb | .400 | .000 | .400 |
|  | Frontal_Med_Orb | .280 | .464 | .280 | Frontal_Mid_Orb | .375 | .250 | .429 |
| **Temporal** | Temporal_Mid | .836 | .655 | .655 | Heschl | .714 | .476 | .190 |
|  | Temporal_Sup | .803 | .662 | .648 | Temporal_Pole_Sup | .588 | .176 | .294 |
|  | Fusiform | .778 | .644 | .600 | Temporal_Inf | .571 | .143 | .286 |
|  | Temporal_Inf | .723 | .660 | .638 | Temporal_Sup | .560 | .360 | .280 |
|  | Hippocampus | .691 | .455 | .527 | Fusiform | .545 | .364 | .273 |
|  | ParaHippocampal | .689 | .533 | .511 | ParaHippocampal | .455 | .364 | .182 |
|  | Temporal_Pole_Sup | .638 | .328 | .552 | Temporal_Mid | .350 | .250 | .250 |
|  | Heschl | .569 | .361 | .431 | Amygdala | .300 | .200 | .300 |
|  | Amygdala | .475 | .169 | .458 | Hippocampus | .294 | .412 | .235 |
|  | Temporal_Pole_Mid | .419 | .323 | .484 | Temporal_Pole_Mid | .111 | .000 | .111 |
| **Parietal** | Paracentralobule | .771 | .457 | .314 | Postcentral | .800 | .640 | .640 |
|  | Postcentral | .733 | .683 | .633 | SupraMarginal | .571 | .476 | .381 |
|  | Parietal_Inf | .642 | .528 | .377 | Angular | .545 | .455 | .091 |
|  | SupraMarginal | .641 | .469 | .359 | Parietal_Inf | .462 | .385 | .231 |
|  | Parietal_Sup | .462 | .538 | .385 | Paracentral_Lobule | .440 | .160 | .360 |
|  | Precuneus | .359 | .156 | .188 | Precuneus | .429 | .238 | .190 |
|  | Angular | .351 | .297 | .216 | Cingulum_Post | .364 | .364 | .091 |
|  | Cingulum_Post | .250 | .063 | .250 |  |  |  |  |
|  | Cuneus | .188 | .125 | .375 |  |  |  |  |
| **Occipital** | Lingual | .611 | .500 | .472 |  |  |  |  |
|  | Occipital_Inf | .571 | .429 | .357 |  |  |  |  |
|  | Occipital_Sup | .500 | .500 | .333 |  |  |  |  |
|  | Occipital_Mid | .417 | .333 | .333 |  |  |  |  |
|  | Calcarine | .250 | .250 | .375 |  |  |  |  |

***Supplementary Table 3:*** *SEEG group probability values during* Speech Arrest *language error according to AAL labels for three frequency bands.* In grey are highlighted the higher probability values (P> .55) observed according to cortical regions in each hemisphere and each lobe.

**Supplementary Table 4**

|  | **SEMANTIC PARAPHASIA** | | | | |
| --- | --- | --- | --- | --- | --- |
|  | **LH** | | | | |
| **LOBE** | **ROI (AAL)** | **70-150 Hz** | **30-70 Hz** | **2-25Hz** |  |
|  | Insula | .700 | .600 | .500 |  |
|  | Rolandic_Oper | .600 | .400 | .500 |  |
|  | Frontal_Mid_Orb | .375 | .250 | .625 |  |
|  | Frontal_Sup_Orb | .375 | .125 | .500 |  |
|  | Frontal_Inf_Orb | .333 | .111 | .667 |  |
|  | Rectus | .250 |  | .375 |  |
|  | Frontal_Med_Orb | .250 |  | .375 |  |
|  | Cingulum_Mid | .111 | .333 |  |  |
|  | Cingulum_Ant | .000 |  | .250 |  |
| **Temporal** | Temporal_Mid | .900 | .800 | .700 |  |
|  | Temporal_Sup | .800 | .600 | .700 |  |
|  | Fusiform | .700 | .700 | .800 |  |
|  | Heschl | .600 | .100 | .200 |  |
|  | Hippocampus | .600 | .400 | .500 |  |
|  | Temporal_Inf | .600 | .600 | .600 |  |
|  | Temporal_Pole_Sup | .500 | .500 | .700 |  |
|  | Temporal_Pole_Mid | .444 | .333 | .556 |  |
|  | ParaHippocampal | .400 | .300 | .500 |  |
|  | Amygdala | .300 | .400 | .500 |  |
| **Parietal** | SupraMarginal | .444 | .222 | .556 |  |
|  | Parietal_Inf | .250 | .375 | .250 |  |
|  | Angular | .222 | .222 | .111 |  |
|  | Precuneus | .200 | .100 | .300 |  |
|  | Cingulum_Post | .111 |  | .444 |  |
| **Occipital** | Lingual | .600 | .400 | .500 |  |

***Supplementary Table 4:*** *SEEG group probability values during* Semantic Paraphasia *language error according to AAL labels for three frequency bands.* In grey are highlighted the higher probability values (P> .55) observed according to cortical regions in each hemisphere and each lobe.

**Supplementary Table 5**

|  | **PHONEMIC PARAPHASIA** | | | | |
| --- | --- | --- | --- | --- | --- |
|  | **LH** | | | | |
| **LOBE** | **ROI (AAL)** | **70-150 Hz** | **30-70 Hz** | **2-25 Hz** |  |
| **Frontal** | Frontal_Inf_Tri | 10 | 10 | .875 |  |
|  | Insula | .909 | .636 | .818 |  |
|  | Frontal_Inf_Oper | .750 | .500 | .625 |  |
|  | Rolandic_Oper | .727 | .455 | .636 |  |
|  | Frontal_Mid_Orb | .667 | .167 | .167 |  |
|  | Frontal_Inf_Orb | .500 | .200 | .300 |  |
|  | Frontal_Med_Orb | .500 | .167 | .167 |  |
|  | Cingulum_Mid | .429 | .286 | .429 |  |
|  | Frontal_Sup_Orb | .400 | .300 | .300 |  |
|  | Cingulum_Ant | .333 | .000 | .167 |  |
|  | Rectus | .333 | .167 | .333 |  |
|  | Precentral | .250 | .125 | .375 |  |
| **Temporal** | Temporal_Mid | 10 | .750 | .875 |  |
|  | Fusiform | .857 | .857 | .714 |  |
|  | Temporal_Inf | .750 | .750 | .750 |  |
|  | Hippocampus | .750 | .500 | .625 |  |
|  | Temporal_Sup | .727 | .455 | .818 |  |
|  | Heschl | .636 | .182 | .636 |  |
|  | ParaHippocampal | .571 | .429 | .571 |  |
|  | Temporal_Pole_Sup | .500 | .375 | .750 |  |
|  | Amygdala | .375 | .375 | .500 |  |
|  | Temporal_Pole_Mid | .286 | .286 | .571 |  |
| **Parietal** | SupraMarginal | .750 | .625 | .750 |  |
|  | Postcentral | .625 | .500 | .875 |  |
| **Occipital** | Occipital_Inf | 10 | .800 | .200 |  |
|  | Lingual | .286 | .286 | .286 |  |

***Supplementary Table 5****: SEEG group probability values during* Phonemic Paraphasia *language error according to AAL labels for three frequency bands.* In grey are highlighted the higher probability values (P> .55) observed according to cortical regions in each hemisphere and each lobe. **Supplementary Table 6**

|  | **NAMING** | | | | | **NAMING** | | | | |
| --- | --- | --- | --- | --- | --- | --- | --- | --- | --- | --- |
|  | **LH** | | | | | **RH** | | | | |
| **LOBE** | **ROI (AAL)** | **70-150 Hz** | **30-70 Hz** | **2-25 Hz** | **ROI (AAL)** | | **70-150 Hz** | **30-70 Hz** | **2-25 Hz** |  |
| **Frontal** | Frontal_Inf_Oper | .850 | .717 | .667 | **Insula** | | **10** | .882 | .500 |  |
|  | Frontal_Inf_Tri | .841 | .873 | .635 | Frontal_Inf_Oper | | .900 | .900 | .533 |  |
|  | Frontal_Mid | .833 | .741 | .648 | Frontal_Mid | | .857 | .524 | .667 |  |
|  | Insula | .781 | .677 | .667 | Supp_Motor_Area | | .810 | .524 | .762 |  |
|  | Supp_Motor_Area | .760 | .720 | .520 | Rectus | | .800 | .000 | .400 |  |
|  | Precentral | .754 | .585 | .631 | Precentral | | .773 | .636 | .727 |  |
|  | Rolandic_Oper | .753 | .591 | .645 | Cingulum_Mid | | .750 | .583 | .667 |  |
|  | Frontal_Sup | .739 | .652 | .565 | Rolandic_Oper | | .710 | .613 | .323 |  |
|  | Frontal_Sup_Medial | .618 | .382 | .218 | Frontal_Inf_Tri | | .636 | .409 | .455 |  |
|  | Frontal_Mid_Orb | .595 | .381 | .429 | Frontal_Sup_Medial | | .611 | .444 | .389 |  |
|  | Frontal_Inf_Orb | .525 | .361 | .426 | Cingulum_Ant | | .524 | .286 | .476 |  |
|  | Cingulum_Mid | .488 | .360 | .500 | Frontal_Sup_Orb | | .500 | .250 | .250 |  |
|  | Frontal_Sup_Orb | .453 | .358 | .358 | Frontal_Sup | | .476 | .238 | .619 |  |
|  | Cingulum_Ant | .351 | .143 | .299 | Frontal_Inf_Orb | | .417 | .250 | .167 |  |
|  | Rectus | .310 | .119 | .262 | Frontal_Med_Orb | | .400 | .000 | .400 |  |
|  | Frontal_Med_Orb | .308 | .128 | .282 | Frontal_Mid_Orb | | .375 | .250 | .250 |  |
| **Temporal** | Temporal_Mid | .863 | .685 | .685 | Fusiform | | .654 | .333 | .250 |  |
|  | Temporal_Sup | .793 | .630 | .674 | ParaHippocampal | | .625 | .333 | .167 |  |
|  | Fusiform | .774 | .677 | .645 | Temporal_Pole_Mid | | .619 | .000 | .111 |  |
|  | Temporal_Inf | .708 | .662 | .646 | Amygdala | | .583 | .143 | .429 |  |
|  | Hippocampus | .685 | .452 | .534 | Temporal_Pole_Sup | | .533 | .238 | .286 |  |
|  | ParaHippocampal | .629 | .484 | .516 | Hippocampus | | .500 | .318 | .273 |  |
|  | Temporal_Pole_Sup | .605 | .355 | .592 | Temporal_Sup | | .333 | .367 | .300 |  |
|  | Heschl | .581 | .312 | .430 | Temporal_Inf | | .320 | .250 | .250 |  |
|  | Amygdala | .442 | .221 | .468 | Heschl | | .273 | .462 | .154 |  |
|  | Temporal_Pole_Mid | .404 | .319 | .511 | Temporal_Mid | | .111 | .240 | .280 |  |
| **Parietal** | Paracentralobule | .737 | .421 | .289 | Postcentral | | .808 | .615 | .615 |  |
|  | Postcentral | .704 | .662 | .648 | SupraMarginal | | .571 | .476 | .381 |  |
|  | SupraMarginal | .630 | .457 | .420 | Angular | | .545 | .455 | .091 |  |
|  | Parietal_Inf | .594 | .516 | .344 | Parietal_Inf | | .462 | .385 | .231 |  |
|  | Parietal_Sup | .429 | .500 | .357 | Paracentral_Lobule | | .440 | .160 | .360 |  |
|  | Angular | .340 | .280 | .200 | Precuneus | | .429 | .238 | .190 |  |
|  | Precuneus | .333 | .141 | .205 | Cingulum_Post | | .364 | .364 | .091 |  |
|  | Cingulum_Post | .230 | .049 | .279 |  | |  |  |  |  |
|  | Cuneus | .150 | .100 | .400 |  | |  |  |  |  |
| **Occipital** | Occipital_Inf | .667 | .524 | .286 |  | |  |  |  |  |
|  | Lingual | .566 | .453 | .453 |  | |  |  |  |  |
|  | Occipital_Sup | .429 | .429 | .286 |  | |  |  |  |  |
|  | Occipital_Mid | .333 | .267 | .267 |  | |  |  |  |  |
|  | Calcarine | .222 | .222 | .333 |  | |  |  |  |  |

***Supplementary Table 6****: SEEG group probability values during* total naming *language error according to AAL labels for three frequency bands.* In grey are highlighted the higher probability values (P> .55) observed according to cortical regions in each hemisphere and each lobe.

**Supplementary Figure 2**

**
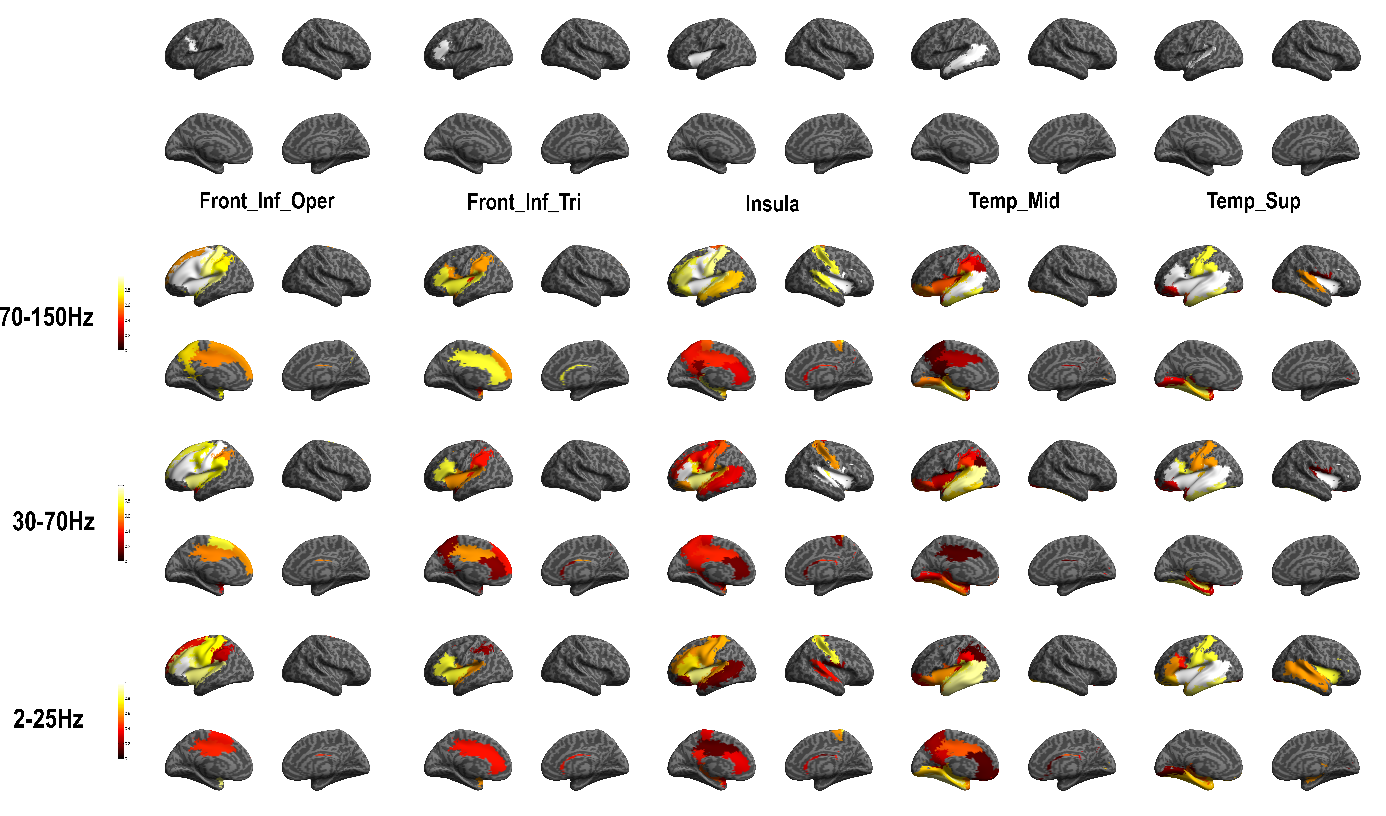
**

***Supplementary Figure 2:*** *SEEG group probability map for each ROI for different frequency bands. Top: anatomical location of ROI (seed) analysis and down: functional sub-network results for each frequency band. The colour bar indicates the ROI-based probability (1: cortical stimulation systematically induced a symptom-related the with specific frequency band increase in this ROI; 0: cortical stimulation never induced a symptom-related with the specific frequency increase in this ROI). Probability values are more important in 70-150Hz band than in the others bands.*

**Supplementary Table 7**

| **Frontal_Inf_Oper** | | | | | | **Frontal_Inf_Tri** | | | | | | **Insula** | | | | | | **Temporal_Mid** | | | | | | **Temporal_Sup** | | | | | |
| --- | --- | --- | --- | --- | --- | --- | --- | --- | --- | --- | --- | --- | --- | --- | --- | --- | --- | --- | --- | --- | --- | --- | --- | --- | --- | --- | --- | --- | --- |
| **ROI (AAL)** | **70-150** | **30-70** | **2-25** | **3-45 AD** | **ROI (AAL)** | | **70-150** | **30-70** | **2-25** | **3-45 AD** | **ROI (AAL)** | | **70-150** | **30-70** | **2-25** | **3-45 AD** | **ROI (AAL)** | | **70-150** | **30-70** | **2-25** | **3-45 AD** | **ROI (AAL)** | | **70-150** | **30-70** | **2-25** | **3-45 AD** |  |
| **Frontal_Inf_Tri_L** | **1** | **1** | **1** | 1 | Cingulum_Ant_L | | .80 | .20 | .40 | .60 | **Insula_L** | | **1** | **.88** | .88 | .88 | **Temporal_Mid_L** | | **1** | **.90** | **.90** | .90 | **Insula_L** | | **1** | **1** | **1** | **1** |  |
| **Rolandic_Oper_L** | **1** | **1** | .88 | 1 | Cingulum_Mid_L | | .80 | .60 | .40 | .80 | **Precentral_L** | | **1** | .38 | .63 | .88 | Fusiform_L | | .90 | .60 | **.90** | .80 | **Temporal_Mid_L** | | **1** | **1** | **1** | **1** |  |
| **Frontal_Inf_Oper_L** | **1** | **1** | .88 | .88 | Frontal_Inf_Tri_L | | .80 | .80 | .80 | .80 | **Rolandic_Oper_L** | | **1** | **.88** | .88 | .88 | Temporal_Inf_L | | .80 | .70 | **.90** | .90 | **Heschl_L** | | **1** | .67 | .67 | .83 |  |
| **Insula_L** | **1** | .88 | .88 | .88 | Insula_L | | .80 | .60 | .80 | .80 | **Rolandic_Oper_R** | | **1** | **1** | .20 | .60 | ParaHippocampal_L | | .70 | .50 | **.70** | .70 | **Rolandic_Oper_L** | | **1** | .83 | .83 | .83 |  |
| **Precentral_L** | **1** | .75 | .75 | .88 | Temporal_Sup_L | | .80 | .60 | .60 | .40 | **Insula_R** | | **1** | **1** | .00 | .20 | Hippocampus_L | | .70 | .40 | **.70** | .80 | **Temporal_Sup_L** | | **1** | **1** | **1** | .83 |  |
| **Frontal_Mid_L** | **1** | **1** | .86 | .86 | Frontal_Inf_Oper_L | | .60 | .80 | .80 | .60 | **Frontal_Inf_Oper_L** | | **1** | **.71** | .71 | .00 | Temporal_Sup_L | | .70 | .70 | **.80** | .80 | **Frontal_Inf_Tri_L** | | **1** | **1** | .60 | .80 |  |
| Postcentral_L | .88 | 1 | .75 | 1 | Frontal_Sup_Medial_L | | .60 | .40 | .00 | .20 | Postcentral_L | | .88 | .50 | .63 | .75 | Frontal_Mid_Orb_L | | .63 | .38 | .38 | .13 | **Frontal_Inf_Oper_L** | | **1** | .80 | .40 | .60 |  |
| Heschl_L | .88 | .75 | .75 | .88 | Parietal_Inf_L | | .60 | .40 | .20 | .60 | Temporal_Sup_L | | .88 | .63 | .63 | .50 | Frontal_Inf_Orb_L | | .56 | .44 | **.56** | .33 | **Insula_R** | | **1** | **1** | .80 | .60 |  |
| Temporal_Sup_L | .88 | .75 | .88 | .75 | Rolandic_Oper_L | | .60 | .60 | .60 | .40 | Frontal_Inf_Tri_L | | .86 | 1 | .71 | .71 | Lingual_L | | .56 | .33 | **.67** | .44 | Fusiform_L | | .83 | .83 | .67 | .83 |  |
| SupraMarginal_L | .75 | .75 | .25 | .50 | SupraMarginal_L | | .60 | .40 | .00 | .40 | Hippocampus_L | | .83 | .33 | .50 | .17 | Frontal_Sup_Orb_L | | .50 | .50 | .38 | .00 | Temporal_Inf_L | | .83 | .83 | .83 | .83 |  |
| Amygdala_L | .75 | .25 | .88 | .25 | Temporal_Pole_Sup_L | | .60 | .20 | .60 | .60 | Frontal_Mid_L | | .80 | .40 | .60 | .80 | Insula_L | | .50 | .30 | **.60** | .40 | Hippocampus_L | | .83 | .67 | .50 | .67 |  |
| Temporal_Pole_Sup_L | .75 | .38 | .88 | .25 | Amygdala_L | | .40 | .00 | .60 | .60 | Postcentral_R | | .80 | .60 | .80 | .60 | Temporal_Pole_Sup_L | | .50 | .30 | **.60** | .50 | Postcentral_L | | .80 | .60 | .80 | .60 |  |
| Parietal_Inf_L | .71 | .57 | .29 | .57 | Heschl_L | | .40 | .40 | .60 | .20 | Temporal_Sup_R | | .80 | **1** | .40 | .60 | SupraMarginal_L | | .44 | .44 | .33 | .56 | SupraMarginal_L | | .80 | .60 | .80 | .60 |  |
| Precuneus_L | .71 | .00 | .00 | .00 |  | |  |  |  |  | Frontal_Inf_Orb_L | | .80 | .60 | .20 | .20 | Rolandic_Oper_L | | .40 | .10 | .50 | .30 | ParaHippocampal_L | | .67 | .33 | .67 | .83 |  |
| Frontal_Sup_L | .60 | .80 | .40 | .80 |  | |  |  |  |  | Heschl_R | | .80 | .80 | .00 | .20 | Parietal_Inf_L | | .33 | .33 | .11 | .56 | Temporal_Sup_R | | .60 | .00 | .60 | .60 |  |
| Supp_Motor_Area_L | .60 | .80 | .40 | .80 |  | |  |  |  |  | Heschl_L | | .75 | .38 | .50 | .25 | Temporal_Pole_Mid_L | | .33 | .00 | **.56** | .56 | Heschl_R | | .60 | .20 | .00 | .00 |  |
| Frontal_Sup_Medial_L | .60 | .60 | .00 | .40 |  | |  |  |  |  | Temporal_Pole_Sup_L | | .67 | .50 | .33 | .83 | Cingulum_Mid_L | | .25 | .13 | .50 | .13 | Temporal_Pole_Sup_L | | .50 | .33 | .67 | .83 |  |
| Cingulum_Mid_L | .57 | .57 | .43 | .71 |  | |  |  |  |  | Amygdala_L | | .67 | .17 | .33 | .33 | Angular_L | | .25 | .13 | .13 | .38 | Amygdala_L | | .33 | .17 | .67 | **1** |  |
|  |  |  |  |  |  | |  |  |  |  | Temporal_Mid_L | | .67 | .33 | .17 | .33 | Amygdala_L | | .22 | .22 | **.56** | .33 | Temporal_Pole_Mid_L | | .33 | .33 | .67 | .67 |  |
|  |  |  |  |  |  | |  |  |  |  | Paracentral_Lobule_R | | .60 | .20 | .60 | .20 | Heschl_L | | .20 | .00 | .30 | .30 | Frontal_Inf_Orb_L | | .33 | .33 | .67 | .50 |  |
|  |  |  |  |  |  | |  |  |  |  | Paracentral_Lobule_L | | .50 | .33 | .33 | .17 | Precuneus_L | | .13 | .00 | .25 | .00 | Lingual_L | | .33 | .00 | .17 | .17 |  |
|  |  |  |  |  |  | |  |  |  |  | Cingulum_Mid_L | | .43 | .43 | .14 | .43 | Cingulum_Post_L | | .11 | .00 | .33 | .00 | Rolandic_Oper_R | | .20 | .20 | .00 | .00 |  |
|  |  |  |  |  |  | |  |  |  |  | Precuneus_L | | .40 | .40 | .00 | .20 |  | |  |  |  |  | Frontal_Sup_Orb_L | | .17 | .33 | .67 | .67 |  |
|  |  |  |  |  |  | |  |  |  |  | Cingulum_Ant_L | | .33 | .17 | .33 | .33 |  | |  |  |  |  | Hippocampus_R | | .00 | .00 | .60 | .60 |  |
|  |  |  |  |  |  | |  |  |  |  | Cingulum_Post_L | | .20 | .40 | .40 | .40 |  | |  |  |  |  | Temporal_Mid_R | | .00 | .00 | .60 | .60 |  |

***Supplementary Table 7****: SEEG group probability values observed during the stimulation on each of the five languages ROI and according to: each of the frequency band explored (frequency of interest HFA 50-150Hz; 30-70Hz, 2-25Hz and 3-45 Hz related with after* *afterdischarge) and AAL labels.* In grey are highlighted the higher probability values (P>.55) and in bold the regions with a P =1*.*
